# Targeting the SPC25/RIOK1/MYH9 Axis to Overcome Tumor Stemness and Platinum Resistance in Epithelial Ovarian Cancer

**DOI:** 10.1002/advs.202406688

**Published:** 2024-11-03

**Authors:** Xingyu Jiang, Muwen Yang, Weijing Zhang, Dongni Shi, Yue Li, Lixin He, Shumei Huang, Boyu Chen, Xuwei Chen, Lingzhi Kong, Yibing Pan, Pinwei Deng, Rui Wang, Ying Ouyang, Xiangfu Chen, Jun Li, Zheng Li, Hequn Zou, Yanna Zhang, Libing Song

**Affiliations:** ^1^ Department of Experimental Research State Key Laboratory of Oncology in South China Collaborative Innovation Center for Cancer Medicine Sun Yat‐sen University Cancer Center Guangzhou Guangdong 510060 China; ^2^ Department of Radiation Oncology Shenzhen Key Laboratory of Gastrointestinal Cancer Translational Research Peking University Shenzhen Hospital Shenzhen Guangdong 518036 China; ^3^ Department of Radiology State Key Laboratory of Oncology in South China Collaborative Innovation Center for Cancer Medicine Sun Yat‐sen University Cancer Center Guangzhou Guangdong 510060 China; ^4^ Department of Biochemistry Zhongshan School of Medicine Sun Yat‐sen University Guangzhou Guangdong 510060 China; ^5^ Department of Pathology State Key Laboratory of Oncology in South China Collaborative Innovation Center for Cancer Medicine Sun Yat‐sen University Cancer Center Guangzhou Guangdong 510060 China; ^6^ Department of Gynecologic Oncology The Third Affiliated Hospital of Kunming Medical University (Yunnan Cancer Hospital) Kunming Yunnan 650118 China; ^7^ School of Medicine The Chinese University of Hong Kong Shenzhen Guangdong 518172 China; ^8^ Department of Gynecology State Key Laboratory of Oncology in South China Collaborative Innovation Center for Cancer Medicine Sun Yat‐sen University Cancer Center Guangzhou Guangdong 510060 China

**Keywords:** cancer stem cell, cell‐penetrating peptide, epithelial ovarian cancer, phosphorylation, platinum resistance, protein‐protein interaction, SPC25

## Abstract

In epithelial ovarian cancer (EOC), platinum resistance, potentially mediated by cancer stem cells (CSCs), often leads to relapse and treatment failure. Here, the role of spindle pole body component 25 (SPC25) as a key determinant promoting stemness and platinum resistance in EOC cells, with its expression being correlated with adverse clinical outcomes is delineated. Mechanistically, SPC25 acts as a scaffolding platform, orchestrating the assembly of an SPC25/RIOK1/MYH9 trimeric complex, triggering RIOK1‐mediated phosphorylation of MYH9 at Ser1943. This prompts MYH9 to disengage from the cytoskeleton, augmenting its nuclear accumulation, thus potentiating *CTNNB1* transcription and subsequent activation of Wnt/β‐catenin signaling. CBP1, a competitive inhibitory peptide, can disrupt the formation of the aforementioned trimeric complex, diminishing the activity of the SPC25/RIOK1/MYH9 axis–mediated Wnt/β‐catenin signaling, and thus attenuate CSC phenotypes, thereby enhancing platinum efficacy in vitro, in vivo, and in patient‐derived organoids. Therefore, targeting the SPC25/RIOK1/MYH9 axis, which mediates the maintenance of stemness and platinum resistance in EOC cells, may enhance platinum sensitivity and increase survival in patients with EOC.

## Graphical abstract

This study demonstrates that SPC25 acts as a molecular scaffold, mediating SPC25/RIOK1/MYH9 complex formation and triggering the RIOK1‐mediated phosphorylation of MYH9 at Ser1943. This disengages MYH9 from the cytoskeleton and increases its nuclear levels in EOC cells, which facilitates the transcriptional regulation of CTNNB1 and activation of Wnt/β‐catenin signaling and thereby induces tumor stemness and platinum resistance. Based on this mechanism, the development of the competitive inhibitory peptide, CBP1, is noted to strongly disrupt the SPC25/RIOK1/MYH9 axis and enhance platinum toxicity in EOC cells.

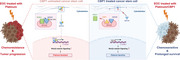

## 1 Introduction

Epithelial ovarian cancer (EOC) is associated with the highest mortality rate among all malignancies of the female reproductive system.^[^ ^1^ ^]^ Despite the inherent heterogeneity among individual tumors, platinum‐based chemotherapy remains the main first‐line adjuvant treatment applied for EOC, enhancing patient survival considerably.^[^ ^2^, ^3^ ^]^ However, approximately 70% of patients with EOC, including those achieving complete response (CR) or partial response (PR) after an adequate number of cycles of platinum‐based chemotherapy, experience relapse or progression within 2 years after treatment.^[^ ^4^, ^5^ ^]^ Furthermore, with successive lines of platinum‐based chemotherapy, EOC tumors progressively become less sensitive to platinum, ultimately leading to treatment failure and patient mortality.^[^ ^6^ ^]^

Cancer stem cells (CSCs), possessing stem cell‐like properties, considerably contribute to tumor growth, metastasis, and recurrence, particularly under therapeutic pressures such as chemotherapy.^[^ ^7^, ^8^, ^9^, ^10^ ^]^ The role of CSCs in EOC progression is critical. Because of an insidious onset and a lack of early screening biomarkers, EOC is diagnosed at advanced stages in most cases, which affords CSCs ample time and space to develop sophisticated drug resistance mechanisms.^[^ ^11^, ^12^ ^]^ Concurrently, the propensity of EOC for peritoneal implantation metastasis and malignant ascites formation alongside a unique, complex peritoneal microenvironment fosters stem‐like property acquisition or enhancement in cancer cells.^[^ ^13^ ^]^ Collectively, CSCs are considered major contributors to the endurance of therapeutic resistance in EOC.^[^ ^14^ ^]^ Therefore, targeting CSCs within EOC tumors may considerably augment platinum‐based drug efficacy or reverse resistance.

Spindle pole body component 25 (SPC25), a constituent of the Ndc80 protein complex, has a crucial role in the cell nucleus division during mitosis.^[^ ^15^ ^]^ Functionally, SPC25 forms a heterodimeric subcomplex with SPC24 and interacts at the kinetochore, mediating accurate chromosome segregation.^[^ ^15^, ^16^ ^]^ Recent studies have highlighted the association of SPC25 with the aggressive progression of various tumors, including recurrence, metastasis, and treatment resistance.^[^ ^17^, ^18^, ^19^ ^]^ Notably, SPC25 overexpression has been noted in a subpopulation of tumor cells exhibiting stem‐like characteristics.^[^ ^19^, ^20^, ^21^ ^]^ However, the precise involvement of SPC25 in the maintenance of stemness and platinum resistance in EOC, along with the specific molecular mechanisms underlying its aberrant function, remain largely unexplored.

Here, we elucidated the key role of SPC25 in mediating the stemness and platinum resistance in EOC and demonstrated a major mechanism whereby the SPC25/RIOK1/MYH9 trimeric complex assembly, mediated by SPC25, triggers Wnt/β‐catenin signaling activation. Notably, CBP1, a competitive inhibitory peptide, was developed to disrupt the formation of this complex in EOC cells and organoids, enhancing their platinum sensitivity. These findings indicate the precise contributions of SPC25 in tumoral stemness and platinum resistance regulation in EOC, suggesting that targeting the SPC25/RIOK1/MYH9 axis is a promising therapeutic strategy to offset platinum resistance in EOC.

## 2 Results

### 2.1 SPC25 Confers Platinum Resistance and Correlates Poor Prognosis in EOC

**Figure 1:**
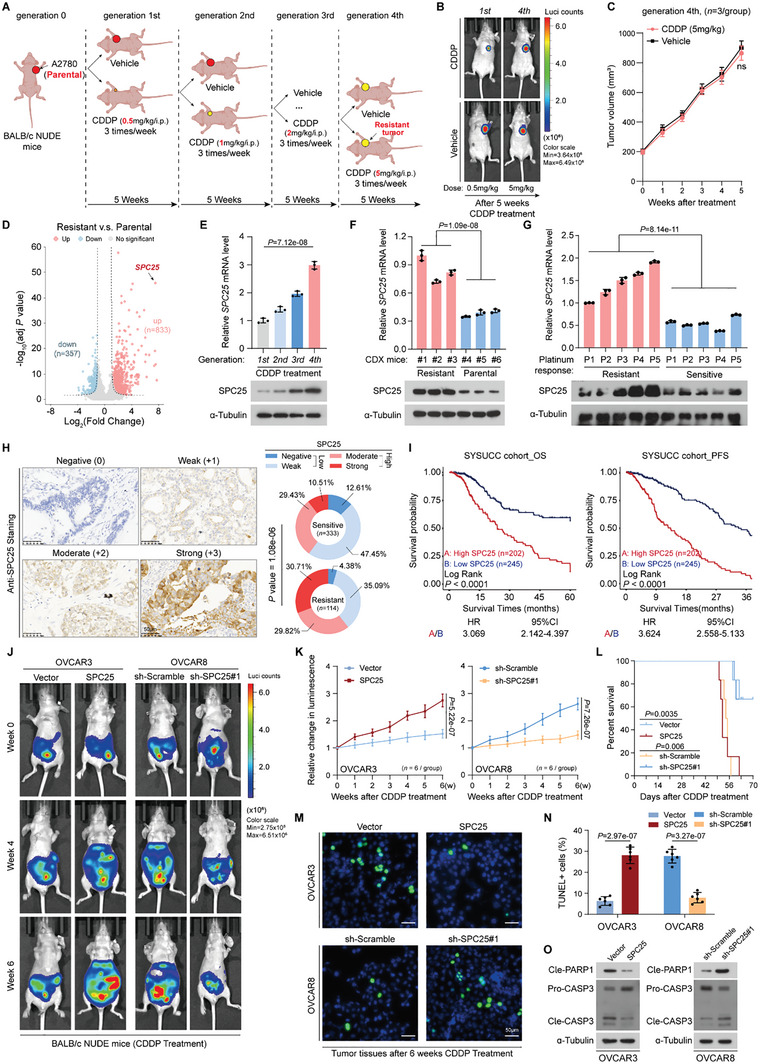
SPC25 confers platinum resistance and is correlated with poor prognosis in EOC. A) Schematic of establishment of CDDP‐resistant xenografted tumor in nude mice with A2780 cells. B) Representative bioluminescence images of nude mice of the first‐ and fourth‐generation in CDDP treatment week 5. C) Curves of volumes of fourth‐generation xenografted tumors calculated in the indicated weeks (*n* = 3 mice per group). D) Volcano plot of genes differentially expressed between the CDDP‐resistant and parental xenografted tumors of A2780 cells. E) *SPC25* expression in xenografted tumors across different generations validated through qRT‐PCR and Western blotting. F) *SPC25* expression in xenografted tumors between the CDDP‐resistant and the parental groups validated through qRT‐PCR and Western blotting (*n* = 3 mice per group). G) *SPC25* expression in 10 fresh clinical EOC tissues with different responses to platinum detected through qRT‐PCR and Western blotting (*n* = 5 samples per group). H) Representative images of IHC staining for SPC25 in 4 EOC tumor specimens, scored as Negative (0), Weak (+1), Moderate (+2), or Strong (+3) (left). Correlation of SPC25 staining and response of platinum‐based adjuvant chemotherapy (right). The chi‐square test was used here. Scale bar, 50 µm. I) Kaplan–Meier survival analysis of patients with EOC stratified by low and high SPC25 expression. Mantel–Haenszel log‐rank test was used here. J) Representative bioluminescence images of CDDP‐treated intraperitoneal tumor‐bearing nude mice in each group in the indicated weeks (*n* = 6 mice per group). K) Relative changes in luminescence signal of intraperitoneal tumors in nude mice receiving CDDP treatment in the indicated weeks. L) Kaplan–Meier survival analysis of intraperitoneal tumor‐bearing nude mice under treatment of CDDP. M,N) Representative images of TUNEL assay in intraperitoneal xenografted tumor tissues (M). Quantification of TUNEL‐positive cells in the indicated groups (N). Scale bar, 50 µm. O) Expression levels of apoptosis‐related proteins in intraperitoneal xenografted tumor tissues assessed through Western blotting. In (E), (F), (G), and (N), *n* = 3 biological replicates. Error bars represent the means ± standard deviations from independent experiments; ns denotes not significant. In (F), (G), and (N), two‐sided Student's *t* test was used. In (E), one‐way analysis of variance was used. In (C) and (K), one‐way repeated‐measures analysis of variance was used.

To ascertain key molecules mediating platinum resistance in EOC, we initiated consecutive passages by administering escalating doses of cisplatin (CDDP) to BALB/c‐Nude mice bearing A2780 human EOC cells (**Figure** **1**A), allowing for the establishment of a CDDP‐resistant EOC model by the fourth generation (Figure 1B,C; Figure S1A,B, Supporting Information). Next, we performed whole‐transcriptome sequencing analysis and identified 1190 genes with differential expression in the CDDP‐resistant group compared with the parental group, comprising 833 upregulated and 357 downregulated genes (Figure 1D). Notably, SPC25 was most considerably upregulated in the CDDP‐resistant group; this aberrant expression was further confirmed through qRT‐PCR and Western blotting in xenograft tumors across different generations (Figure 1E,F). Moreover, high SPC25 expression was consistently observed across transcriptome datasets from various platinum‐resistant EOC cell lines, including SKOV3, OVCAR5, OVCAR4, PEA1, and PEO1 (Figure S1C, Supporting Information). To assess SPC25 expression in clinical samples, we examined 10 fresh EOC tissues, categorizing them into platinum‐sensitive and ‐resistant groups according to their clinical response to platinum‐based chemotherapy. The findings revealed a substantial upregulation of SPC25 expression in the platinum‐resistant clinical tumor tissue samples, corroborating our earlier preclinical observations (Figure 1G). To investigate the clinical relevance of SPC25 further, we assessed its expression in tissue sections from 447 EOC cases that had undergone platinum‐based chemotherapy (Table S1, Supporting Information). Similarly, we observed increases in SPC25 expression in the specimens of patients with platinum‐resistant EOC (Figure 1H); this finding was corroborated by external data from The Cancer Genome Atlas (TCGA) (Figure S1D, Supporting Information). Our Kaplan–Meier analysis revealed that high SPC25 expression is strongly associated with reduced overall survival (OS) and progression‐free survival (PFS) rates (Figure 1I; Figure S1E, Supporting Information). Furthermore, we included SPC25 expression along with other factors that could potentially influence the prognosis of EOC patients—such as tumor differentiation, FIGO stage, pathological subtype, and treatment regimen—in a multivariate Cox regression analysis, and the results showed that SPC25 expression remained significantly associated with poor prognosis (Figure S2A,B, Supporting Information). To account for differences in prognosis due to varying FIGO stages and pathological subtypes, we also performed multivariate analyses within different FIGO stages (Stage I, II, III, and IV) and across distinct pathological subtypes (serous, mucinous, endometrioid, and clear cell). The results consistently indicated that SPC25 expression is an independent risk factor for poor prognosis in chemotherapy‐treated EOC patients (Figure S2C,B and S3A,B, Supporting Information). Taken together, the aforementioned results suggest that SPC25 acts as a key mediator of resistance to platinum‐based chemotherapy in EOC.

Subsequently, we selected 4 representative EOC cell lines (Figure S1F,G, Supporting Information) and validated the role of SPC25 in mediating platinum resistance in EOC through a series of in vitro and in vivo experiments. As shown in Figure S1H–K (Supporting Information), SPC25 overexpression significantly promoted growth in EOC cells with inherently low SPC25 levels, including OVCAR3 and COV644 cells, in the presence of CDDP; it also considerably reduced CDDP‐induced apoptosis. In contrast, SPC25 silencing enhanced CDDP sensitivity in EOC cells with inherently high SPC25 levels, such as OVCAR8 and SKOV3 cells. To further establish an EOC mouse model, we intraperitoneally injected BALB/c‐Nude mice with EOC cells with stable luciferase expression. CDDP treatment (three times per week, a week as a cycle) was initiated when the bioluminescence signal reached 2 × 10^7 ^ p/s/cm^2^/sr. After 6 weeks of continuous treatment cycles, as demonstrated by the representative in vivo imaging of mice (Figure 1J) and the corresponding statistical data (Figure 1K), OVCAR3 cells with SPC25 overexpression demonstrated a notable increase in CDDP resistance (Figure S1L,M, Supporting Information). This was also indicated by decreased survival of tumor‐bearing mice (Figure 1L), reduced number of terminal deoxynucleotidyl transferase dUTP nick end labeling (TUNEL)‐positive cells, and lowered activation of caspase‐3 and poly (ADP‐ribose) polymerase (PARP; Figure 1M–O). In contrast, SPC25 silencing in OVCAR8 cells led to an increase in CDDP sensitivity and apoptotic index, as well as prolonged survival, in the tumor‐bearing mice (Figure 1J–O; Figure S1L,M, Supporting Information). Taken together, these results suggested that SPC25 plays a pivotal role in promoting resistance to platinum‐based chemotherapy in EOC tumors and is associated with unfavorable prognostic outcomes.

### 2.2 SPC25‐Induced Transcriptional Regulation of *CTNNB1* Activates Wnt/β‐Catenin Signaling and Stemness in EOC Cells

**Figure 2:**
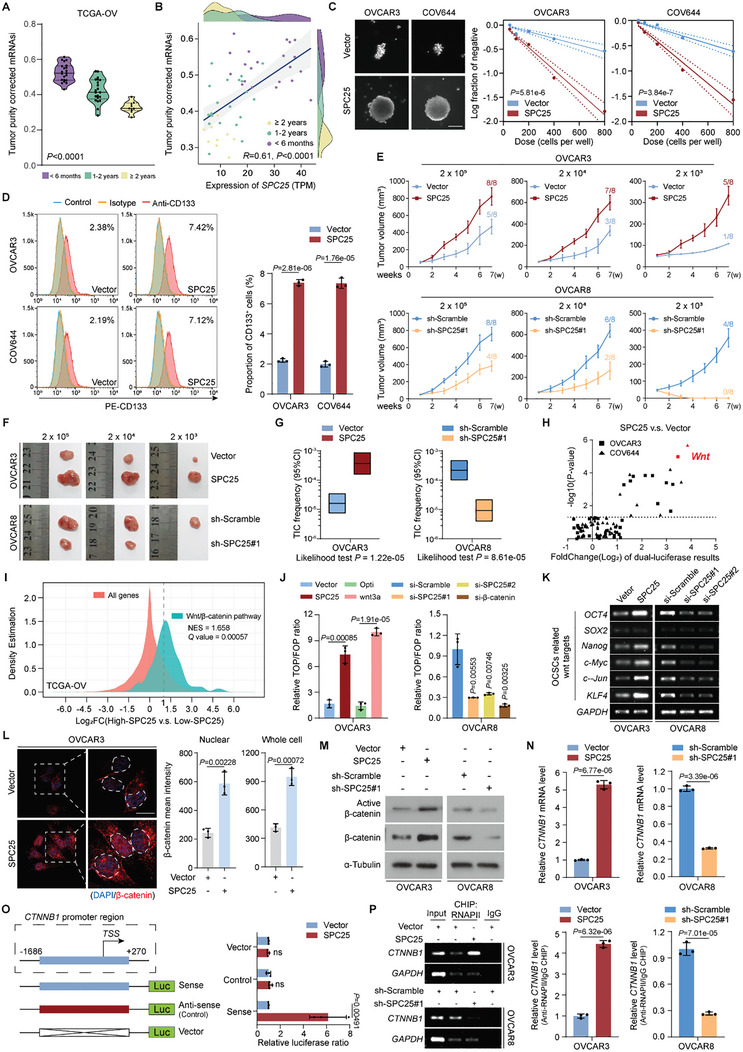
SPC25‐induced transcriptional regulation of *CTNNB1* activates Wnt/β‐catenin signaling and stemness in EOC cells. A) Violin plot of tumor purity–corrected mRNAsi in different tumor progression‐free survival time groups. B) Correlation between *SPC25* expression and tumor purity–corrected mRNAsi. Spearman correlation analysis was used here. C) Representative images of tumor‐spheres formation the limitation dilution assays of OVCAR3 and COV644 cells with *SPC25* overexpression (left). Log‐dose slope of the vector and SPC25 overexpression groups (right). Scale bar, 200 µm. D) Percentage of CD133‐positive subpopulations from the indicated OVCAR3 and COV644 cells. E,F) Limiting dilution assays of the indicated OVCAR3 and OVCAR8 cells. Control, *SPC25*‐overexpressing, and *SPC25*‐silenced tumor cells were inoculated subcutaneously at 2 × 10^5^, 2 × 10^4^, and 2 × 10^3^ into nude mice (*n* = 8 per group; E). Representative images of tumors from indicated groups; each minor division on the scale of the ruler represents 1 mm (F). G) Frequency of tumor‐initiating cells in the indicated groups. H) Cignal Finder Reporter Array for signal transduction 45 pathway showing that *SPC25* overexpression significantly activated Wnt/β‐catenin signaling in EOC cells. I) Density plot demonstrating the GSEA results. J) Relative ratio of luciferase activities of TOP‐Flash over FOP‐Flash normalized using Renilla luciferase activity in OVCAR3 and OVCAR8 cells with the indicated treatment (Wnt‐3a, 1.6 ng/mL, overnight). K) qRT‐PCR of OCSC‐related Wnt target genes *OCT4*, *SOX2*, *Nanog*, *c‐Myc*, *c‐Jun*, and *KLF4* in control, *SPC25*‐overexpressing, and *SPC25*‐silenced EOC cells. L) Immunofluorescence of β‐catenin expression in control and *SPC25*‐overexpressing OVCAR3 cells (left). Quantification of nuclear or cellular fluorescence (right). Scale bar, 10 µm. M) Expression levels of β‐catenin and activated β‐catenin in OVCAR3 and OVCAR8 cells with the indicated treatments assessed through Western blotting. N) qRT‐PCR of *CTNNB1* in *SPC25*‐overexpressing or *SPC25*‐silenced OVCAR3 and OVCAR8 cells. O) Schematic illustration of the sense or antisense *CTNNB1* promoter regions cloned into the pGL3 luciferase reporter plasmid (left). Quantification of luciferase activity of the *CTNNB1* promoter‐reporter in the indicated cells (right). P) RIP assays followed by qRT‐PCR to examine interactions between RNAPII and *CTNNB1* mRNAs in control, *SPC25*‐overexpressing, and *SPC25*‐silenced EOC cells. *GAPDH* was used as the negative control. In (D), (J), (L), (N), (O), and (P), *n* = 3 biological replicates. Error bars represent the means ± standard deviations from independent experiments; ns denotes not significant. In (D), (J), (L), (N), (O), and (P), a two‐sided Student's *t*‐test was used. In (A), a one‐way analysis of variance was used. In (C), a likelihood ratio test was used.

CSCs are major contributors to chemotherapy resistance and tumor relapse. We initially used Gene Set Enrichment Analysis (GSEA) based on sequencing data from CDDP‐resistant and parental xenograft tumors (Figure 1) and noted substantial enrichment of stem cell‐related pathways in the CDDP‐resistant group (Figure S4A, Supporting Information). Further analysis of TCGA data revealed a significant increase in tumor purity–corrected mRNA stemness index (mRNAsi; adjusted for) in EOC cells,^[^ ^22^ ^]^ which was inversely correlated with PFS after platinum‐based chemotherapy (**Figure** **2**A). This result highlighted the major role of CSCs in mediating treatment failure in EOC. Moreover, we noted a marked positive correlation between SPC25 expression and tumor purity–corrected mRNAsi (Figure 2B). Thus, SPC25 may play a crucial role in promoting stemness in tumor cells, potentially contributing to resistance to platinum‐based chemotherapy.

Supporting this hypothesis, limitation dilution assays revealed that SPC25 overexpression significantly enhanced self‐renewal and tumorigenic properties in OVCAR3 and COV644 cells. In contrast, SPC25 silencing considerably diminished stem‐like traits in OVCAR8 and SKOV3 cells (Figure 2C; Figure S4B, Supporting Information). Upregulating SPC25 expression significantly increased the abundance of the CD133‐positive subpopulations of OVCAR3 and COV644 cells, whereas downregulating SPC25 markedly reduced that of CD133‐positive subpopulations of OVCAR8 and SKOV3 cells (Figure 2D; Figure S4C, Supporting Information). These findings suggested that SPC25 promotes stemness in EOC cells in vitro. We further validated the tumor‐initiating ability of SPC25 in vivo through limiting dilution assays in BALB/c‐Nude mice. After administering 2 × 10^5^, 2 × 10^4^, and 2 × 10^3^ SPC25‐overexpressing OVCAR3 cells, we observed tumor formation in mice at 7 weeks after injection in almost all treated mice (8/8, 7/8, and 5/8, respectively). This result was in contrast to that noted for the control groups, whereby tumors developed in significantly fewer mice (5/8, 3/8, and 1/8, respectively; Figure 2E). Similarly, administration of 2 × 10^3^ OVCAR8‐shSPC25#1 cells led to no tumor development in mice, whereas that of 2 × 10^5^ and 2 × 10^4^ OVCAR8‐shSPC25#1 cells resulted in few tumors. In contrast, 2 × 10^5^, 2 × 10^4^, and 2 × 10^3^ control OVCAR8 cells induced tumor formation in 8, 6, and 4 of 8 mice, respectively (Figure 2E,F). Further analysis revealed that SPC25 expression significantly increases the abundance of tumor‐initiating cells (TICs) within tumors, whereas its suppression reduces it considerably (Figure 2G). Therefore, SPC25 may be crucial in maintaining EOC cell stemness.

To elucidate the molecular mechanism underlying SPC25‐mediated promotion of the stem cell properties in EOC cells, we used OVCAR3 and COV644 cells and employed a Cignal Finder Reporter Array Plate for signal transduction 45 pathway to probe the downstream signaling perturbations induced by SPC25 overexpression. Our findings revealed a marked activation of the canonical Wnt/β‐catenin signaling cascade subsequent to SPC25 overexpression in both OVCAR3 and COV644 cells (Figure 2H). Furthermore, GSEA of sequencing data from xenograft tumors and TCGA data demonstrated significant enrichment of the Wnt/β‐catenin pathway in both CDDP‐resistant xenograft tumors and SPC25‐overexpressing EOC cases (Figure 2I; Figure S4D, Supporting Information). Similarly, TOP/FOP Flash reporter assays for further validation demonstrated that SPC25 overexpression enhanced luciferase activity, whereas SPC25 silencing decreased it (Figure 2J; Figure S4E, Supporting Information). Moreover, in both OVCAR3 and OVCAR8 cells, as well as their mouse xenograft tumors, we observed significant changes in the expression of downstream target genes of the Wnt/β‐catenin pathway, including those associated with ovarian cancer stem cells (OCSCs) after SPC25 overexpression or knockdown (Figure 2K; Figure S4F,G, Supporting Information). Notably, XAV939, a specific antagonist of the canonical Wnt pathway, effectively mitigated the enhanced stemness attributable to SPC25 overexpression, suggesting that SPC25 exerts its effects via the activation of the canonical Wnt/β‐catenin signaling axis (Figure S4H,I, Supporting Information).

Further immunofluorescence (IF) analysis revealed that SPC25 overexpression not only facilitates the nuclear accumulation of β‐catenin in OVCAR3 cells but also amplifies the global fluorescent signal of β‐catenin across the cellular milieu (Figure 2L). Thus, SPC25 may have a regulatory role in the modulation of β‐catenin expression. In further validation through Western blotting, SPC25 elevation was noted to markedly augment β‐catenin levels (Figure 2M). Consequently, we posit that SPC25 orchestrates the modulation of Wnt/β‐catenin signaling through its effects on regulating β‐catenin expression. Nevertheless, cycloheximide (CHX) chase assays and RNA immunoprecipitation (RIP)‐qPCR targeting eukaryotic translation initiation factor 4E (eIF4E) elucidated that SPC25 expression did not affect the stability or translational efficiency of β‐catenin in both OVCAR3 and OVCAR8 cells (Figure S4J–M, Supporting Information). Moreover, our actinomycin D (Act D) assays delineated that the effects of SPC25 do not affect the half‐life of *CTNNB1* mRNA (Figure S4N,O, Supporting Information). We also discovered that SPC25 expression significantly altered *CTNNB1* mRNA levels (Figure 2N). In particular, luciferase reporter assays and chromatin immunoprecipitation (ChIP)‐qPCR targeting RNA Polymerase II (RNAPII) revealed that SPC25 substantially fosters the transcriptional activation of *CTNNB1* (Figure 2O,P). Taken together, these results indicated that SPC25 increases β‐catenin expression at the transcriptional level, thereby activating the Wnt/β‐catenin signaling and inducing stemness in EOC cells.

### 2.3 SPC25 Regulates *CTNNB1* Transcription Dependent on Its Interaction with MYH9 in the Cytoplasm

**Figure 3:**
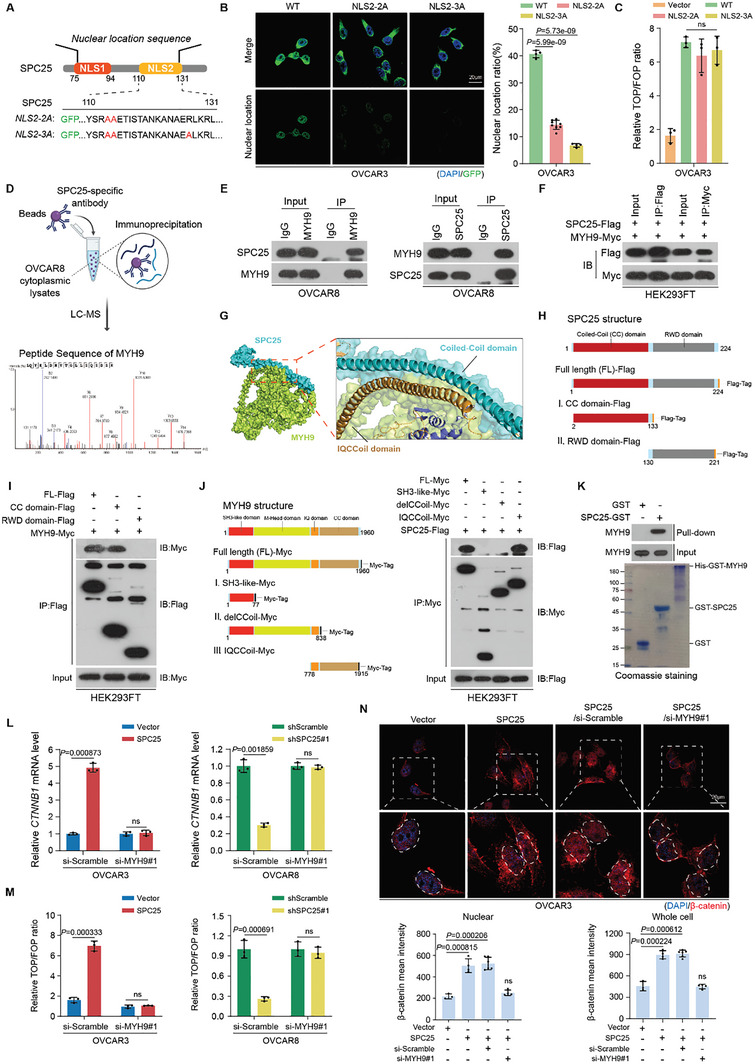
SPC25 regulates *CTNNB1* transcription dependent on its interaction with MYH9 in the cytoplasm. A) Schematic of the predicted NLSs within the SPC25 protein sequence, including NLS1 and NLS2. The NLS2 sequences of NLS2‐2A (two‐point mutations) and NLS2‐3A (three‐point mutations); the red letters indicate critical sites in SPC25 NLS2. B) Fluorescence of GFP‐tagged SPC25 with NLS2‐2A and NLS2‐3A mutants in OVCAR3 cells (left). Quantitation of the ratio of the nuclear GFP luminescence (right; 4–10 GFP‐positive cells counted under 60× field of view). Scale bar, 20 µm. C) Relative ratio of luciferase activities of TOP‐Flash over FOP‐Flash normalized using Renilla luciferase activity in OVCAR3 cells transfected with WT, NLS2‐2A, and NLS2‐3A mutants of *SPC25*. D) Co‐IP–MS analysis of the SPC25 interactome in cytoplasmic lysates of OVCAR8 cells by using anti‐SPC25. E) Endogenous interaction between SPC25 and MYH9 validated using IP assays in OVCAR8 cells. F) Exogenous interaction between SPC25 and MYH9 was validated through IP assays in HEK293FT cells transfected with SPC25‐Flag and MYH9‐Myc constructs. G) Structure model of the protein complex composed of SPC25 and MYH9 monomers predicted using AlphaFold2. H) Schematic of SPC25 protein truncated constructs. I) HEK293FT cells transfected with indicated MYH9‐Myc and SPC25‐Flag truncations, followed by IP assays with Flag‐beads, for assessing their detailed interactions with MYH9. J) Schematic illustration of MYH9 protein truncated constructs (left). HEK293FT cells transfected with indicated SPC25‐Flag and MYH9‐Myc truncations, followed by IP assays with Myc‐beads, to assess their detailed interactions with SPC25. K) In vitro GST‐pulldown assays with purified SPC25 and MYH9 (upper). Purified SPC25 and MYH9 validated through SDS‐PAGE and Coomassie Blue staining (lower). L) qRT‐PCR of *CTNNB1* in control or *MYH9*‐silenced OVCAR3 or OVCAR8 cells, followed by *SPC25* overexpression or silencing. M) TOP/FOP Flash analysis in control or *MYH9*‐silenced OVCAR3 or OVCAR8 cells, followed by *SPC25* overexpression or silencing. N) IF of β‐catenin expression in control or *MYH9*‐silenced OVCAR3 cells with or without *SPC25* overexpression (upper). Quantification of nuclear or cellular fluorescence (lower). Scale bar, 20 µm. In (B), (C), (L), (M), and (N), *n* = 3 biological replicates. Error bars represent the means ± standard deviations from independent experiments; ns denotes not significant. In (B), (L), (M), and (N), a two‐sided Student's *t*‐test was used. In (C), a one‐way analysis of variance was used.

SPC25, an essential component of the Ndc80 complex, plays a critical role in mitosis.^[^ ^15^ ^]^ Hence, in addition to being localized in the cytoplasm, SPC25 is markedly enriched in the nucleus according to the human gene database data (Figure S5A, Supporting Information).^[^ ^23^ ^]^ To further elucidate the role of SPC25 in modulating *CTNNB1* transcriptional activity, we initially focused on the impact of the subcellular localization of SPC25. In particular, we engineered mutations targeting its nuclear localization sequences (NLSs) predicted by NLStradamus,^[^ ^24^ ^]^ namely NLS1 and NLS2, to produce NLS‐2A and NLS‐3A mutants (**Figure** **3**A; Figure S5B, Supporting Information). Notably, the NLS2‐3A mutant exhibited a significant reduction in the nuclear localization of GFP‐tagged SPC25 (Figure 3B; Figure S5C, Supporting Information). Subsequent functional assays demonstrated that inhibiting nuclear localization of SPC25 had no discernible effect on the activation of the Wnt/β‐catenin pathway or the expression of *CTNNB1* (Figure 3C; Figure S5D, Supporting Information). Thus, we believe that the transcriptional regulatory role of SPC25 on *CTNNB1* primarily occurs in the cytoplasm rather than the nucleus. Furthermore, in the analysis of cytoplasmic lysates of OVCAR8 cells through liquid chromatography (LC)–mass spectrometry (MS), we identified MYH9, also called nonmuscle myosin IIA (NMIIA), as significantly enriched among all proteins potentially interacting with SPC25 (Figure 3D). To verify the relationship between MYH9 and SPC25, we initially assessed their interaction through both endogenous and exogenous immunoprecipitation (IP) assays (Figure 3E,F). To further delineate the details of this interaction, we employed AlphaFold2 to predict the possible complex structure formed by SPC25 and MYH9 monomers (Figure 3G).^[^ ^25^ ^]^ Thereafter, we generated truncated variants of both SPC25 and MYH9 for further IP experiments. Our findings revealed that only SPC25 truncations with the coiled‐coil (CC) domain can interact with MYH9. In contrast, the interactions of MYH9 with SPC25 are restricted to truncations incorporating both the IQ and CC domains (i.e., the IQCC domain; Figure 3H–J). Therefore, the CC domain of SPC25 and the IQCC domain of MYH9 are essential for the SPC25–MYH9 interaction, corroborating the AlphaFold2 predictions. We also verified this direct interaction by using a prokaryotic expression system to produce purified SPC25 and MYH9 proteins and then performing in vitro GST‐pulldown assays (Figure 3K).

To clarify the role of MYH9 in SPC25‐mediated activation of the Wnt/β‐catenin pathway, we silenced MYH9 in OVCAR3 or OVCAR8 cells (Figure S5E, Supporting Information) and investigated its influence on Wnt signaling, modulated by SPC25 expression. The results demonstrated that silencing MYH9 markedly diminished both the transcriptional activation of *CTNNB1* and the subsequent activation of Wnt/β‐catenin signaling triggered by SPC25 expression (Figure 3L,M). Furthermore, the IF results demonstrated that silencing MYH9 substantially decreased the cytoplasmic and nuclear β‐catenin signaling induced by SPC25, indicating that MYH9 is essential for the induction of the effects of SPC25 (Figure 3N). Moreover, our analysis of TCGA transcriptome data revealed a significant positive correlation among *SPC25*, *MYH9*, and *CTNNB1* expression (Figure S5F,G, Supporting Information). However, this correlation diminished after the expression levels of either *SPC25* or *MYH9* were adjusted (Figure S5F,G, Supporting Information), confirming that SPC25‐mediated activation of *CTNNB1* transcription and Wnt/β‐catenin signaling is dependent on MYH9.

### 2.4 SPC25‐Mediated SPC25/RIOK1/MYH9 Complex Formation Enables MYH9 Phosphorylation at Ser1943

**Figure 4:**
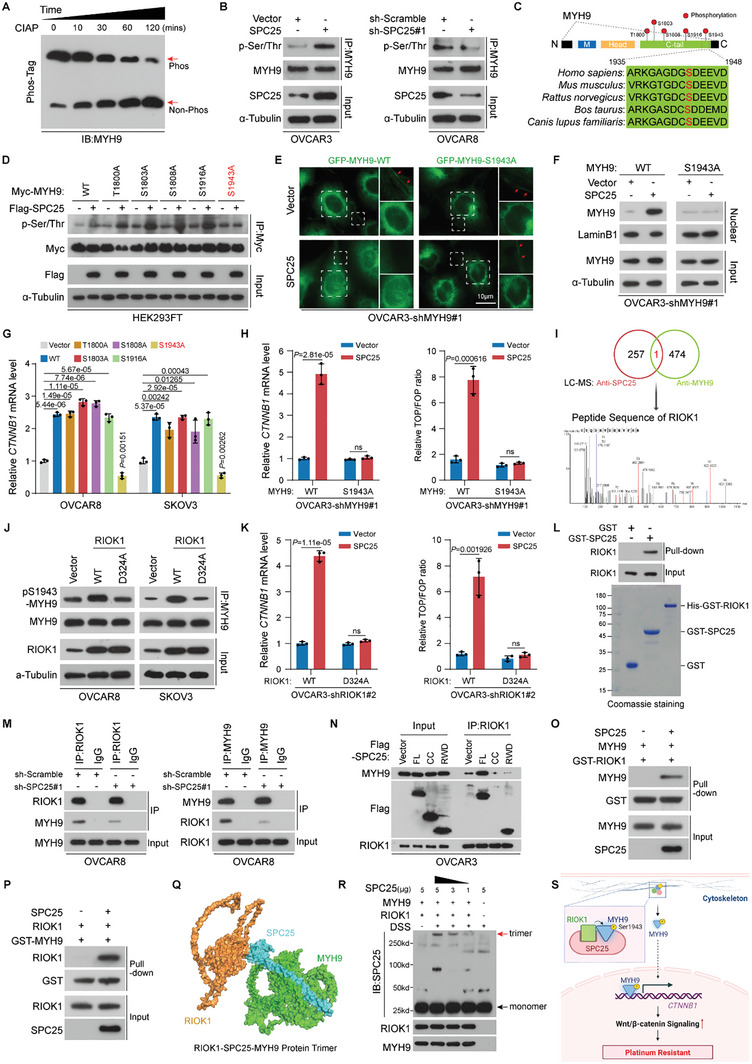
SPC25‐mediated formation of SPC25/RIOK1/MYH9 trimeric complex enables phosphorylation of MYH9 at Ser1943. A) Western blotting of proteins extracted from OVCAR8 cells and subjected to Phos‐tag SDS‐PAGE. B) IP assays to assess the Ser/Thr phosphorylation status of MYH9 in OVCAR3 and OVCAR8 cells in the indicated groups. C) Schematic of the Ser/Thr phosphorylation sites within CT of MYH9. Conserved sequences of MYH9‐Ser1943 across different species are indicated. D) IP assays to assess the Ser/Thr phosphorylation status of MYH9 in HEK293FT cells after the introduction of SPC25‐Flag and variants of MYH9‐Myc mutants. E) Fluorescence of reexpressed GFP‐MYH9‐WT with NLS2‐2A and NLS2‐3A mutants in *MYH9*‐silenced OVCAR3 cells with or without *SPC25* overexpression. Scale bar, 10 µm. F) Western blotting for changes in MYH9 levels in nuclear extraction fractions and total cell lysates. G) qRT‐PCR of *CTNNB1* in OVCAR8 and SKOV3 cells with exogenous expression of variants of MYH9 mutants. H) qRT‐PCR analysis of *CTNNB1* in *MYH9*‐silenced OVCAR3 cells with reexpression of *MYH9*‐WT or *MYH9*‐S1943A with or without *SPC25* overexpression (left). TOP/FOP Flash analysis in *MYH9*‐silenced OVCAR3 cells with reexpression of *MYH9*‐WT or *MYH9*‐S1943A with or without *SPC25* overexpression (right). I) Co‐IP/MS of the MYH9 interactome in cytoplasmic lysates of OVCAR8 cells by using anti‐MYH9. Venn plot indicated the potential protein interacting with both SPC25 and MYH9. J) Phosphorylation levels of MYH9 at Ser1943 in OVCAR8 and SKOV3 cells with exogenous expression of RIOK1‐WT and RIOK1‐D324A. K) qRT‐PCR of *CTNNB1* in *RIOK1*‐silenced OVCAR3 cells with reexpression of *RIOK1*‐WT or *RIOK1*‐D324A with *SPC25* overexpression (left). TOP/FOP Flash analysis in *RIOK1*‐silenced OVCAR3 cells with reexpression of *RIOK1*‐WT or *RIOK1*‐D324A with *SPC25* overexpression (right). L) In vitro GST‐pulldown assays with purified SPC25 and RIOK1 protein (upper). Purified SPC25 and RIOK1 were examined through SDS‐PAGE and Coomassie Blue staining (lower). M) Endogenous interactions between RIOK1 and MYH9 validated through IP assays in control or *SPC25*‐silenced OVCAR8 cells. N) IP assays of RIOK1 in OVCAR3 cells with exogenous expression of full‐length or truncated Flag‐tagged SPC25 constructs. O,P) In vitro GST‐pulldown assays of purified RIOK1 and MYH9 with the presence or absence of purified SPC25. Q) Structural model of protein complex composed of SPC25, MYH9, and RIOK1 monomers predicted using AlphaFold2. R) In vitro crosslinking of purified SPC25, MYH9, and RIOK1 with disuccinimidyl suberate, followed by Western blotting with anti‐SPC25. S) Schematic of mechanisms underlying SPC25‐mediated platinum resistance in EOC cells. In (G), (H), and (K), *n* = 3 biological replicates. Error bars represent the means ± standard deviations from independent experiments; ns denotes not significant. In (G), (H), and (K), a two‐sided Student's *t*‐test was used.

The phosphorylation of serine/threonine (Ser/Thr) residues in the C‐terminal (CT) region of MYH9 is a pivotal determinant of its functional heterogeneity.^[^ ^26^ ^]^ By using phosphate‐affinity SDS‐PAGE (Phos‐tag), we probed OVCAR8 cell lysates and noted an additional slow‐migrating signal of MYH9, which progressively declined after exposure to calf intestinal alkaline phosphatase (CIAP) treatment (**Figure** **4**A). Subsequently, IP experiments revealed that SPC25 expression significantly alters Ser/Thr phosphorylation levels in MYH9 in OVCAR3, OVCAR8, and HEK293FT cells (Figure 4B; Figure S6A, Supporting Information), implicating SPC25 as a modulator of the phosphorylation status of MYH9. Furthermore, we engineered alanine mutants at phosphorylation sites in the CT region of MYH9, specifically Thr1800, Ser1803, Ser1808, Ser1916, and Ser1943—all of which modulate the biological roles of MYH9 (Figure 4C). Notably, our IP results indicated that except for the Ser1943Ala variant (MYH9‐S1943A), all mutants, including the wild‐type MYH9 (MYH9‐WT), exhibited enhanced Ser/Thr phosphorylation in the presence of SPC25, suggesting that SPC25 expression can enhance phosphorylation of MYH9 at Ser1943 (Figure 4D), which was also conserved among different species (Figure 4C).

MYH9 is a crucial component of the cytoskeleton, and phosphorylation at its Ser1943 promotes dissociation from actin filaments and dispersal throughout the cytoplasm and nucleus.^[^ ^26^, ^27^ ^]^ MYH9 within the nucleus can act as a transcriptional regulatory factor, controlling the expression of various downstream genes, including *CTNNB1* and thereby contributing to the malignant progression of tumors.^[^ ^28^, ^29^, ^30^, ^31^ ^]^ As such, we posited that in EOC cells, after SPC25‐mediated phosphorylation at its Ser1943, MYH9 may disengage from the cytoskeleton and function as a transcriptional regulatory factor within the nucleus. To validate this hypothesis, we expressed both GFP‐tagged MYH9‐WT and MYH9‐S1943A in MYH9‐silenced OVCAR3 cells. Fluorescence microscopy revealed that SPC25 overexpression led to reduced localization of GFP‐MYH9‐WT in the cytoskeleton and its notable enrichment in the nucleus without altering the overall fluorescence signal significantly; in SPC25 expression did not affect the subcellular localization of GFP‐MYH9‐S1943A (Figure 4E; Figure S6B, Supporting Information). Similarly, Western blotting of nuclear fractions from MYH9‐silenced OVCAR3 cells confirmed the role of SPC25 in increasing nuclear MYH9 levels, independent of its total expression (Figure 4F). Moreover, to explore whether the phosphorylation of MYH9 at Ser1943 activates *CTNNB1* transcription in EOC cells, we expressed various MYH9 mutants in OVCAR8 or SKOV3 cells and assessed *CTNNB1* mRNA levels; the results demonstrated that only MYH9‐S1943A diminished *CTNNB1* expression (Figure 4G; Figure S6C, Supporting Information), possibly because of the mutant competitively inhibited endogenous MYH9 function. Notably, compared with that of MYH9‐WT, the reintroduction of MYH9‐S1943A in MYH9‐silenced OVCAR3 or OVCAR8 cells impeded the SPC25‐induced *CTNNB1* transcription and Wnt/β‐catenin signaling activation (Figure 4H; Figure S6D,E, Supporting Information). Taken together, these results suggested that SPC25 increases MYH9 phosphorylation at Ser1943, enhancing the nuclear accumulation of MYH9 and consequently modulating the transcription of *CTNNB1*.

Given the nonkinase nature of SPC25, we assessed the involvement of casein kinase II (CKII), which phosphorylates MYH9 at Ser1943,^[^ ^32^, ^33^ ^]^ in the regulatory mechanism of SPC25. However, we noted that SPC25‐mediated promotion of MYH9 phosphorylation at Ser1943 persisted even when kinase activity of endogenous CKII was completely inhibited using the specific inhibitor CX‐4945, indicating the presence of an alternative phosphorylation pathway mediated by SPC25 (Figure S6F,G, Supporting Information). Therefore, we performed LC‐MS analysis on cytoplasmic lysates from OVCAR8 cells and identified potential kinases involved in SPC25‐promoted MYH9 phosphorylation, with only RIOK1 emerging as a likely candidate kinase for SPC25–MYH9 interaction (Figure 4I). To verify whether RIOK1 mediates MYH9 phosphorylation at Ser1943, we experimentally expressed both wild‐type RIOK1 (RIOK1‐WT) and the kinase‐inactive mutant RIOK1‐D324A in OVCAR8 and SKOV3 cells. Moreover, Western blotting revealed that compared with RIOK1‐D324A, RIOK1‐WT significantly increased the expression of MYH9 phosphorylated at Ser1943 (Figure 4J). Furthermore, silencing RIOK1 in OVCAR3 and OVCAR8 cells, followed by reintroduction of RIOK1‐WT and RIOK1‐D324A, revealed that compared with RIOK1‐WT, RIOK1‐D324A significantly diminished SPC25‐mediated *CTNNB1* transcription and Wnt/β‐catenin signaling activation (Figure 4K; Figure S6H—J, Supporting Information). These findings highlighted that SPC25‐driven phosphorylation of MYH9 at Ser1943 depends on the presence of RIOK1.

To elucidate the interplay among SPC25, MYH9, and RIOK1 further, we initially confirmed the interaction between SPC25 and RIOK1 via both endogenous and exogenous protein IP assays (Figure S6K,L, Supporting Information). Leveraging AlphaFold2 predictions of the protein complex structure of SPC25 and RIOK1 monomers (Figure S6M, Supporting Information), we constructed RIOK1 truncated variants for subsequent IP assays. These experiments revealed that the RWD domain of SPC25 and the CT domain of RIOK1 containing the protein kinase (PK) functional area are essential for SPC25–RIOK1 interaction, corroborating the AlphaFold2 predictions (Figure S6N,O, Supporting Information). Simultaneously, we obtained purified RIOK1 via a prokaryotic expression system and confirmed the similarly direct interaction between SPC25 and RIOK1 by using extracellular GST‐pulldown assays (Figure 4L). However, the specific mediating role of SPC25 in RIOK1‐mediated phosphorylation of MYH9 remains unclear. Notably, our IP experiments revealed that the RIOK1–MYH9 interaction becomes significantly weakened after SPC25 knockdown in OVCAR8 cells (Figure 4M). Similarly, in OVCAR3 cells, only full‐length (FL) Flag‐tagged SPC25, among other truncated variants, enhanced the RIOK1–MYH9 interaction and activated Wnt/β‐catenin signaling (Figure 4N; Figure S6P,Q, Supporting Information). This finding was supported by the results of our extracellular GST‐pulldown assays, suggesting that SPC25 acts as a molecular scaffold facilitating the interaction between RIOK1 and MYH9, enabling RIOK1‐mediated phosphorylation of MYH9 (Figure 4O,P). However, the type of complex formed by the SPC25–MYH9–RIOK1 interaction requires clarification. We first predicted the complex structure formed by the monomers of these 3 proteins using AlphaFold2. These predictions were noted to align with our experimental findings, indicating that SPC25 binds directly with both MYH9 and RIOK1, but no direct interaction occurs between RIOK1 and MYH9 (Figure 4Q). To investigate heterooligomeric protein formation further, we performed extracellular pull‐down assays with the purified proteins of all 3 components in the presence of disuccinimidyl suberate (DSS). The results demonstrated that the concentrations of only the SPC25/RIOK1/MYH9 trimeric complex increased with an increase in SPC25 levels in the binding system (Figure 4R).

Taken together, these findings indicated that as a molecular scaffold, SPC25 directs the formation of the SPC25/RIOK1/MYH9 trimeric complex and mediates RIOK1‐mediated phosphorylation of MYH9 at Ser1943. This phosphorylation leads to the release of MYH9 from the cytoskeleton and an increase in its nuclear concentration, facilitating *CTNNB1* transcription and Wnt/β‐catenin signaling activation (Figure 4S).

### 2.5 Disturbing SPC25–RIOK1 Interactions via CBP1 Enhances CDDP Efficacy in EOC

**Figure 5:**
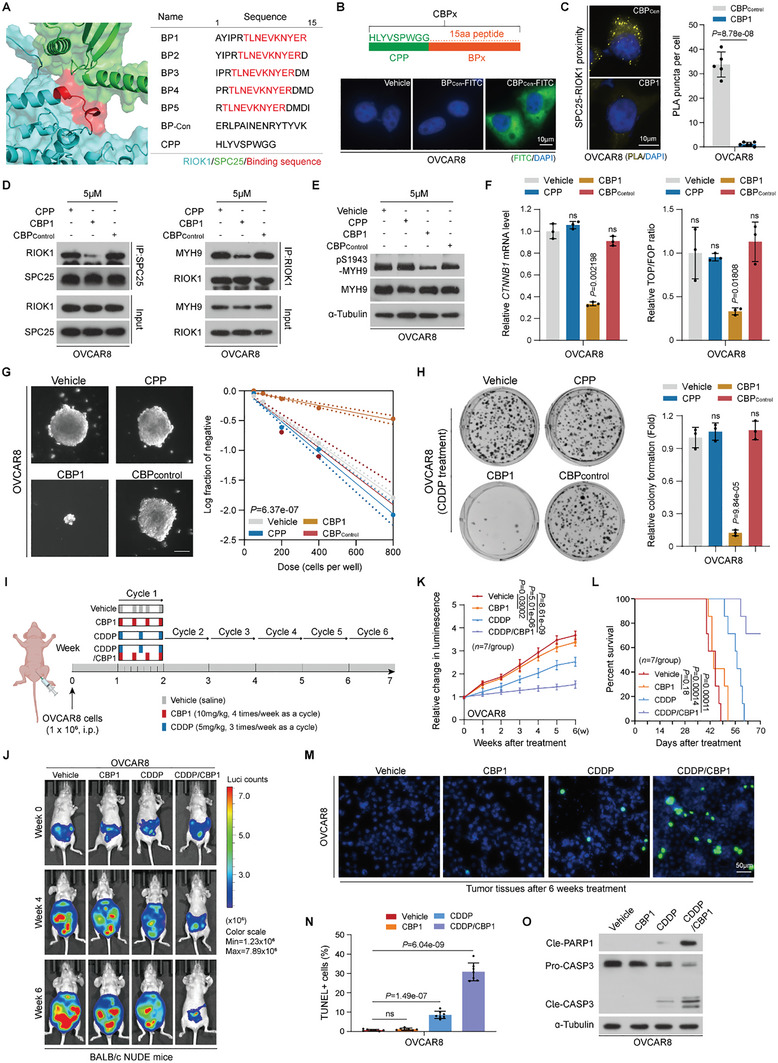
Disturbing the SPC25–RIOK1 interaction via CBP1 enhances CDDP treatment efficacy in EOC. A) Secondary structure of SPC25 and RIOK1 predicted using AlphaFold2 (left). Amino acid (aa) sequences of α‐helical peptides covering SPC25‐RIOK1 binding region from RIOK1 are shown (right). B) Schematic of the fusion peptide (upper). Intracellular delivery of indicated peptides examined through fluorescence microscopy (lower). Scale bar, 10 µm. C) Representative images and quantification of PLA signals indicating SPC25‐RIOK1 interaction in OVCAR8 cells treated with CBP_control_ or CBP1. The PLA signal was quantified by counting the foci per cell from 5 random fields. Scale bar, 10 µm. D) Endogenous interaction between RIOK1 and SPC25 (left panel) or MYH9 (right panel) validated through IP assays in OVCAR8 cells treated with the indicated peptides. E) Phosphorylation levels of MYH9 at Ser1943 in OVCAR8 cells treated with the indicated peptides examined through Western blotting. F) qRT‐PCR of *CTNNB1* in OVCAR8 cells treated with the indicated peptides (left). TOP/FOP Flash analysis in OVCAR8 cells treated with the indicated peptides (right). G) Representative images of tumor‐spheres formation the limitation dilution assays of OVCAR8 cells with indicated treatment (left). Log‐dose slope of indicated treatment groups (right). Scale bar, 200 µm. H) Representative images of surviving colonies of OVCAR8 cells treated with CDDP (10 µM) along with the indicated peptides treatment (left). Quantification of surviving colonies (right). I) Schematic of OVCAR8 cell inoculation in nude mice and schedule of treatment in different groups. J) Representative bioluminescence images of intraperitoneal tumor‐bearing nude mice in each treatment group in the indicated weeks (*n* = 7 mice per group). K) Relative changes in luminescence signal of intraperitoneal tumors in nude mice receiving the indicated treatments in the indicated weeks. L) Kaplan–Meier survival analysis of intraperitoneal tumor‐bearing nude mice after the indicated treatments. M,N) Representative images of TUNEL assay in intraperitoneal xenografted tumor tissues (M). Quantification of TUNEL‐positive cells from the indicated treatment groups (N). Scale bar, 50 µm. O) Expression of apoptosis‐related proteins in intraperitoneal xenografted tumor tissues assessed through Western blotting. In (C), (F), (H), and (N), *n* = 3 biological replicates. Error bars represent the means ± standard deviations from independent experiments; ns denotes not significant. In (C), (F), and (H), a two‐sided Student's *t*‐test was used. In (K), a one‐way repeated‐measures analysis of variance was used. In (G), a likelihood ratio test was used.

Considering the nonconsensus motif‐independent kinase activity of the atypical kinase RIOK1, along with the extensive, complex binding interface between SPC25 and MYH9, we designed 5 small peptides (i.e., BP1, BP2, BP3, BP4, and BP5) based on the AlphaFold2‐predicted complex model of the SPC25 and RIOK1 interaction sites (**Figure** **5**A). To evaluate the potential of these peptides to disrupt the SPC25–RIOK1 interaction in EOC cells, we engineered each peptide with a 9 amino acid cell‐penetrating peptide (CPP) at the N‐terminus,^[^ ^34^ ^]^ generating the CBPx fusion peptides and optimizing their intracellular delivery (Figure 5B). Proximity ligation assay (PLA) revealed that compared with control peptides, only CBP1 most significantly inhibited the SPC25–RIOK1 interaction in OVCAR8 cells (Figure 5C; Figure S7A, Supporting Information). Preliminary in vitro screenings also demonstrated that the presence of CBP1 affected enhancing the sensitivity of OVCAR8 cells to CDDP most substantially (Figure S7B, Supporting Information). In addition, surface plasmon resonance (SPR) analysis of the interaction between CBP1 and SPC25 demonstrated that CBP1 displayed a high affinity for SPC25 (dissociation constant Kd = 13.29 ± 2.88 µM) (Figure S7C, Supporting Information); thus, we selected CBP1 for further investigation. Co‐IP assays confirmed that at 5 µM, CBP1 significantly impeded the interaction between SPC25 and RIOK1, concurrently weakening the interaction between RIOK1 and its substrate MYH9 (Figure 5D; Figure S7D, Supporting Information). Western blotting revealed that CBP1 notably decreased the phosphorylation levels of MYH9 at Ser1943, suppressed *CTNNB1* expression, and inhibited Wnt/β‐catenin signaling activation (Figure 5E,F; Figure S7E, Supporting Information). The functional cellular experiment results revealed that in OVCAR8 cells, CBP1 significantly hindered self‐renewal and tumorigenic capabilities and markedly enhanced the inhibitory effect of CDDP (Figure 5G,H).

We next investigated the safety profile of CBP1 and its therapeutic potential when combined with CDDP as a treatment strategy for EOC in vivo. We initially treated OVCAR8 tumor‐bearing mice with a range of drug concentrations and found that CBP1 exhibited maximal tumor inhibition when administered at a dose of 10 mg/kg (Figure S7F, Supporting Information). In addition, we administered CBP1 (10 mg/kg, once every 2 days) intraperitoneally to BALB/c‐Nude mice not bearing tumors or other concurrent drugs administered over 6 weeks and observed that high‐dose CBP1 did not cause considerable hepatorenal toxicity or affect survival in the mice (Figure S7G,H, Supporting Information), indicating the safety of CBP1. Subsequently, we established a BALB/c‐Nude mouse model of EOC by intraperitoneally injecting OVCAR8 cells stably expressing a luciferase gene, as described previously. On reaching a bioluminescence signal of 2 × 10^7^ p/s/cm^2^/sr, mice were divided into 4 treatment groups: Vehicle, CBP1, CDDP, and CDDP/CBP1 (*n* = 7 per group; Figure 5I). After 6 weeks of continuous treatment cycles, as demonstrated by the representative in vivo imaging of mice (Figure 5J) and the corresponding statistical data (Figure 5K), the CBP1 alone marginally inhibited tumor proliferation (Figure S7I, Supporting Information), likely because of its negative effects on tumor self‐renewal and initiation capacities. Notably, the combination of CDDP and CBP1 significantly enhanced CDDP sensitivity, as evidenced by increased tumor growth inhibition and apoptosis in OVCAR8 cells compared to CDDP alone, effectively prolonging mouse survival (Figure 5J–O; Figure S7I, Supporting Information). These results validated the critical role of the interactions among SPC25, MYH9, and RIOK1 in CDDP resistance in EOC, indicating that targeting the SPC25/RIOK1/MYH9 axis can inhibit tumor cell stemness and that it yields a potent anticancer efficacy when combined with CDDP.

### 2.6 Validating the Clinical Viability of Cisplatin/CBP1 Combination Strategy in EOC Patients‐Derived Organoids

**Figure 6:**
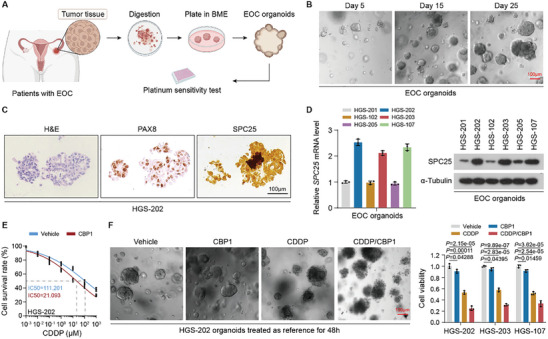
Validating the clinical viability of CDDP/CBP1 combination strategy in organoids derived from patients with EOC. A) Schematic of EOC organoid development. B) Representative images of organoid growth recorded through bright‐field microscopy. Scale bar, 100 µm. C) Hematoxylin–eosin and IHC staining for PAX8 and SPC25 in serial organoid sections. Scale bar, 100 µm. D) Expression of SPC25 across the indicated organoids assessed through qRT‐PCR (left) and Western blotting (right). E) Cell Counting Kit‐8 assay showing that cell viability was significantly impaired in organoids after CDDP treatment in the presence of CBP1. F) Representative images of organoids in the indicated treatment groups (left). Cell viability of each treatment group was detected through CellTiter‐Glo 3D assay (right). Scale bar, 100 µm. In (D), (E), and (F), *n* = 3 biological replicates. Error bars represent the means ± standard deviations from independent experiments. In (E), a one‐way repeated‐measures analysis of variance was used. In (F), a two‐sided Student's *t*‐test was used.

Organoids derived from patient tissues provide a more accurate assessment of drug efficacy, with results reflecting the clinical settings more effectively than those of animal experiments.^[^ ^35^ ^]^ Therefore, we established 6 organoids from different tissues from patients with high‐grade serous ovarian cancer—one of the most lethal pathological subtypes of EOC. The process of tumor tissue processing and organoid culture is depicted in **Figure** **6**A. In the evaluation of the organoids, we first observed the organoid growth daily after revival and performed histological identification through immunohistochemical (IHC) staining (Figure 6B,C; Figure S8A, Supporting Information). We also examined SPC25 expression in all 6 organoids and selected HGS‐202, HGS‐203, and HGS‐107 for further experimentation, all of which demonstrated relatively high SPC25 expression (Figure 6D). We observed that, compared with the controls, the presence of CBP1 markedly enhanced CDDP cytotoxicity in these EOC organoids (Figure 6E; Figure S8B, Supporting Information). Furthermore, we divided the organoids into 4 treatment groups: Vehicle, CBP1, CDDP, and CDDP/CBP1 (Figure 6F). Next, we assessed cell viability using the CellTiter‐Glo Luminescent assay 48 h after treatment. Similar to the in vivo results, CBP1 significantly increased the response to CDDP in the EOC organoids (Figure 6F; Figure S8C, Supporting Information). Notably, patients from whom these 3 EOC organoids were obtained were all clinically assessed as platinum tolerant during subsequent first‐line platinum‐based chemotherapy. Therefore, CBP1 has a strong clinical potential as a sensitizer or reversal agent for platinum resistance during EOC treatment.

### 2.7 Clinical Relevance of SPC25 and Nuclear MYH9 in EOC Patients

**Figure 7:**
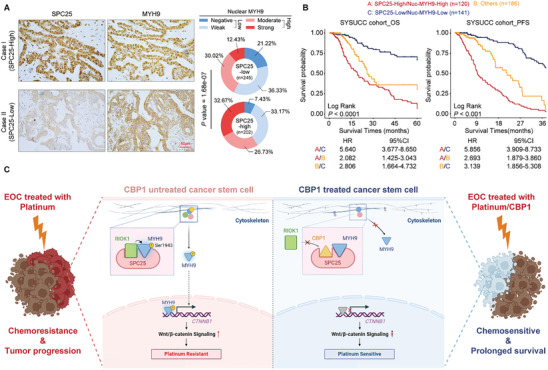
Clinical relevance of SPC25 and nuclear MYH9 in patients with EOC. A) Representative images of IHC staining for SPC25 and MYH9 in human EOC specimens (left). Correlation of SPC25 expression levels with nuclear MYH9 levels (right) in 447 human EOC specimens based on the chi‐square test. Scale bar, 50 µm. B) Kaplan–Meier analysis for OS and PFS compared in groups with high SPC25 and nuclear MYH9 levels, low SPC25 and nuclear MYH9 levels, and others. C) Schematic of the role of the SPC25/RIOK1/MYH9 axis in the maintenance of stemness and platinum resistance in EOC. The competitive inhibitory peptide CBP1 combined with CDDP has potent anticancer efficacy.

We finally assessed the clinical relevance and significance of the SPC25/MYH9 axis in patients with EOC. Notably, IHC staining and subsequent correlation analyses revealed a significant positive correlation between SPC25 expression and nuclear MYH9 level in 447 specimens from patients with EOC (**Figure** **7**A; Table S1, Supporting Information). Notably, our Kaplan–Meier survival curves demonstrated that patients with high SPC25 expression and high nuclear MYH9 accumulation had the poorest 5‐year OS and PFS among patients with EOC (Figure 7B).

In summary, our results demonstrated that SPC25 acts as a molecular scaffold, mediating SPC25/RIOK1/MYH9 complex formation and triggering the RIOK1‐mediated phosphorylation of MYH9 at Ser1943. This disengages MYH9 from the cytoskeleton and increases its nuclear levels in EOC cells, which facilitates the transcriptional regulation of *CTNNB1* and activation of Wnt/β‐catenin signaling and thereby induces tumor stemness and platinum resistance. Based on this mechanism, the development of the competitive inhibitory peptide, CBP1, was noted to strongly disrupt the SPC25/RIOK1/MYH9 axis and enhance platinum toxicity in EOC cells (Figure 7C).

## 3 Discussion

CSCs enhance cancer cell survival by augmenting DNA damage repair and apoptotic resistance, particularly under therapeutic pressures, including chemotherapy, radiotherapy, and targeted treatments.^[^ ^36^, ^37^, ^38^, ^39^ ^]^ Moreover, CSCs demonstrate metabolic plasticity, adapting energy pathways to thrive in metabolically compromised environments caused by low oxygen and nutrient deficiencies due to therapeutic stress.^[^ ^40^, ^41^ ^]^ Furthermore, CSCs engage with the tumor microenvironment by interacting with immune cells and fibroblasts and emulating microenvironmental components (e.g., tumor vasculogenic mimicry) so as to bolster tumor resilience against therapy.^[^ ^42^, ^43^, ^44^ ^]^ Considering the presence of these multifaceted resistance mechanisms, direct targeting of CSC genesis may be more efficacious than addressing individual resistance pathways. However, currently available CSC‐directed therapy is associated with several limitations. For instance, targeting specific CSC markers, such as CD44 and CD133, are insufficiently specific or lose effectiveness over time because of the heterogeneity and dynamism of CSC marker expression patterns.^[^ ^45^, ^46^ ^]^ Furthermore, the dependence of CSC on key signaling pathways such as the Notch, Wnt, and Hedgehog pathways for stemness maintenance complicates therapeutic targeting.^[^ ^47^ ^]^ Direct pathway inhibition often leads to resistance via signaling crosstalk and pathway dominance variances across tumor types.^[^ ^48^, ^49^ ^]^ Moreover, these pathways are often crucial in normal stem cells as well, indicating concerns regarding the toxicity of chemotherapeutics in normal tissues.^[^ ^50^, ^51^ ^]^ Therefore, therapeutic strategies targeting the signal sources, leading to aberrant pathway activation within specific tumor entities and malignancy profiles, might be relatively effective. In the current study, we identified SPC25 as specifically upregulated in platinum‐resistant EOC, correlating with poor prognosis. Our findings revealed that SPC25 overexpression mediates a function distinct from its physiological role by facilitating the assembly of the SPC25/RIOK1/MYH9 trimeric complex, thereby regulating *CTNNB1* transcription and activating Wnt/β‐catenin signaling, culminating in the promotion of CSC properties. Notably, disrupting the interaction of the trimeric complex mediated by SPC25 or direct targeting of SPC25 via shRNA‐mediated degradation markedly sensitizes EOC tumors to platinum. Hence, targeting the SPC25/RIOK1/MYH9 axis may be a novel strategy to counteract platinum resistance.

SPC25, involved in the formation of the Ndc80 complex, mediates the interaction between microtubules and centromeres, ensuring accurate mitotic processes.^[^ ^15^, ^16^ ^]^ Moreover, SPC25, overexpressed in various malignancies, is related to poor prognosis.^[^ ^17^, ^52^, ^53^ ^]^ However, the molecular mechanisms underlying the nontraditional physiological functions of SPC25, particularly in the mediation of tumor malignancy progression, have not been reported. Therefore, our findings indicated, for the first time, that SPC25 operates as a scaffolding protein, fostering the novel trimeric complex SPC25/RIOK1/MYH9 and activating the canonical Wnt/β‐catenin pathway. Notably, a previous study indicated that SPC25 can also act as a downstream gene of β‐catenin, initiating its expression simultaneously with Wnt signaling activation. A positive feedback loop comprising SPC25 and β‐catenin may also exist in EOC cells; it illustrates the intricacies of Wnt pathway signal transduction in tumor cells and highlights SPC25 as a central target within the positive feedback circuitry. These findings demonstrated that SPC25 may not be a mere byproduct of Wnt/β‐catenin signal transduction; rather, it may play a crucial role in Wnt signaling activation in EOC cells.

RIOK1, a RIO kinase family member and an atypical Ser/Thr PK, is conserved in higher eukaryotes.^[^ ^54^ ^]^ In contrast to classical PKs, including those from the AGC kinase family, atypical kinases such as RIOK1 can phosphorylate substrates in the absence of specific sequence or motif recognition, thereby displaying a diverse range of substrate specificities and biological functions.^[^ ^54^, ^55^, ^56^ ^]^ Nevertheless, the precise mechanism underlying substrate phosphorylation by RIOK1, independent of specific sequence motifs, warrants elucidation. SPC25‐mediated trimeric complex formation is requisite for the MYH9 phosphorylation activity of RIOK1, indicating that RIOK1 is located proximal to its substrates; this promotes the catalytic function of RIOK1. Therefore, our result revealed, for the first time, a molecular mechanism by which an atypical kinase achieves catalytic action, providing reference and support for subsequent research.

MYH9 is a pivotal component of the cytoskeleton and plays crucial roles in cell migration, division, and morphology maintenance.^[^ ^26^ ^]^ MYH9 may exert oncogenic potential through diverse signaling pathways and molecular mechanisms, including β‐catenin homeostasis modulation at both protein and transcriptional levels.^[^ ^30^, ^57^, ^58^ ^]^ However, several studies, including those on EOC, have mainly characterized MYH9 as a downstream effector molecule rather than a driving factor, exercising relevant functions after they receive upstream signals. Similarly, our findings demonstrated that although MYH9 expression does not significantly differ between platinum‐sensitive and ‐resistant EOC tissues, it can be the executor molecule responding to SPC25 upregulation signal, facilitating the transcriptional activation of β‐catenin. Consequently, our results underscored the significance of identifying and targeting upstream regulators as a therapeutic strategy, particularly for molecules such as MYH9 that maintain essential biological functions.

Currently, the efficacy of combining platinum‐based chemotherapy with biological agents has been validated through long‐term clinical practice and trials related to EOC. For instance, adding bevacizumab to platinum‐based regimens can prolong PFS in recurrent patients with EOC by 3–4 months compared with controls.^[^ ^59^ ^]^ However, the short extension of PFS and a lack of significant improvement in OS have limited the applicability of this combined approach.^[^ ^60^ ^]^ Furthermore, the use of PARP inhibitors (e.g., olaparib and niraparib) during chemotherapy yields marginal PFS or OS enhancements; however, these inhibitors have demonstrated marked benefits in the maintenance treatment of EOC for patient survival.^[^ ^61^, ^62^, ^63^ ^]^ Notably, the effectiveness of PARP inhibitors decreases with successive treatment lines, which reduces the PFS gain to approximately 3 months.^[^ ^64^ ^]^ Therefore, the development of newer clinical therapeutics to meet the patients’ treatment needs is warranted. In the current study, we newly synthesized the small molecule peptide CBP1 and demonstrated its potential as a platinum sensitizer in preclinical models. Nevertheless, the translation of CBP1 to clinical application necessitates further efficacy, safety, and bioavailability validation in subsequent studies.

## 4 Experimental Section

### Cell Culture

The human EOC cell lines SKOV3 and OVCAR3 were obtained from the American Type Culture Collection (Manassas, VA, USA) and cultured in McCoy's 5A medium containing 10% fetal bovine serum (FBS) at 37 °C in a 5% CO_2_ atmosphere. Another human EOC cell line, OVCAR8, was purchased from Hysigen Bioscience (Suzhou, China) and cultured in Roswell Park Memorial Institute (RPMI)‐1640 medium (Thermo Scientific, Waltham, MA, USA) containing 10% FBS at 37 °C in a 5% CO_2_ atmosphere. The EOC cell lines COV644 and A2780 were purchased from ECACC and cultured in RPMI‐1640 medium containing 10% FBS at 37 °C in a 5% CO_2_ atmosphere. HEY‐A8 (human EOC cell line) and HEK293FT (human embryonic kidney cell line) were kindly gifted by Dr. Li Jun at the Department of Biochemistry, Zhongshan School of Medicine, Sun Yat‐sen University (Guangzhou, China) and maintained in Dulbecco's modified Eagle's medium (DMEM) containing 10% FBS at 37 °C in a 5% CO_2_ atmosphere. All cells were assessed based on via short tandem repeat (STRs) to confirm that they were negative for *Mycoplasma* contamination.

### Xenograft Tumor Models and Treatments

Female BALB/c‐Nude mice (age = 4–5 weeks) were purchased from GemPharmatech (Guangdong, China) and housed in barrier facilities under a 12‐h light/dark cycle. For the establishment of CDDP‐induced resistant tumor models, phosphate‐buffered saline (PBS) suspensions containing the A2780‐luciferase cells (1 × 10^6^) were injected subcutaneously into mice at day 0. Tumor growth monitoring was performed weekly using an IVIS Spectrum optical imaging system (PerkinElmer); the tumor volume was calculated as (length × width^2^)/2. Tumors were harvested when they became palpable and then minced into small pieces (≤2 mm in size). The mince tumors were subsequently mixed with 5 mL of digestion medium [comprising 500 µL of collagenase/hyaluronidase (#07912; Stemcell Technologies), 750 µL of DNase I solution (#10104159001; Roche), and 3.75 mL of RPMI‐1640 medium] at 37 °C for 20 min on a shaking platform to obtain single‐cell suspensions. These suspensions were then mashed through 40‐mm filters into RPMI‐1640, and the filtrate was centrifuged at 300 × g at 4 °C for 5 mins. The supernatant was discarded, and 10 mL of ammonium chloride solution (#A9434; Sigma Aldrich) was added to the precipitate to remove erythrocytes at RT for 5 min, followed by centrifugation at 300 × g for 5 min. The supernatant was subsequently discarded, and tumor cells were resuspended in PBS at 1 × 10^6^ cells/mL. Next, 1 mL of this suspension was injected subcutaneously into each mouse as the first generation. After 1 week of growth, the mice were randomly divided into 2 groups (*n* = 3 per group), and CDDP (0.5 mg/kg in PBS; #HY‐17394; MedChem Express) or vehicle (1× PBS) was administered intraperitoneally to these 2 groups 3 times per week for up to 5 weeks. At week 5, the regrown tumors were dissociated and passaged to the next mice using the same steps mentioned above and treated with the vehicle or CDDP (1.0 mg/kg in PBS). As shown in Figure 1A, consecutive passages were conducted similarly with escalating doses of CDDP (with 5 mg/kg in the fourth generation). The fourth‐generation tumors were excised for RNA sequencing, qRT‐PCR, and Western blotting.

For limiting dilution assays, 2 × 10^5^, 2 × 10^4^, or 2 × 10^3^ control OVCAR3, SPC25‐overexpressing OVCAR3, control OVCAR8, or OVCAR8‐shSPC25#1 cells were mixed with Matrigel (BD Biosciences, San Jose, CA, USA) and then injected subcutaneously into mice. Tumor‐free mouse percentages and tumor volumes were analyzed; each minor division on the scale of the ruler in Figure 2F represents 1 mm. The TIC frequency was calculated using the ELDA (http://bioinf.wehi.edu.au/software/elda/).

In the intraperitoneal tumor model, 1 × 10^6^ of the indicated cells stably expressing luciferase were injected intraperitoneally into the mice. Treatment with saline (Vehicle), CDDP (5 mg/kg, 3 times per week for 1 week), CBP1 (10 mg/kg, once in 2 days), or a combination of CDDP (5 mg/kg, 3 times per week for 1 week) and CBP1 (10 mg/kg, once in 2 days) was initiated when the bioluminescence signal reached 2 × 10^7^ p/s/cm^2^/sr for 6 weeks of continuous treatment cycles. The mice were sacrificed when they became moribund, as determined by an observer blinded to the treatment. Finally, their tumors were excised for qRT‐PCR and Western blotting.

### Clinical Specimens

Paraffin‐embedded specimens of tumor tissues from 447 patients histopathologically diagnosed as having EOC and treated with platinum‐based adjuvant chemotherapy postsurgically at the Sun Yat‐sen University Cancer Center over 2004–2024. Table S1 (Supporting Information) summarizes the clinicopathological characteristics of the patients, including the details of chemotherapy.

### Patient‐Derived Organoids Culture and Treatments

Fresh surgical EOC tumor tissues were obtained from the Sun Yat‐sen University Cancer Center. Before sampling, written informed consent was obtained from all patients. Tissue processing and organoid culture procedures were performed as indicated in Figure 6A. In brief, freshly excised tumor tissues were immediately digested using 2 mg mL^−1^ collagenase (#C9407; Sigma Aldrich) at 37 °C for 12 h, and the dissociated cells were coated in growth factor‐reduced Matrigel (#356231; Corning). This was followed by Matrigel–cell solution solidification in 48‐well plates and then by immediate administration of EOC organoid medium (Accurate International Biotech); this medium was refreshed every 2–3 days. Organoids were collected for qRT‐PCR and Western blotting after 3–4 weeks of culture. Subsequently, organoids were subcultured approximately every 14–21 days. For drug sensitivity testing, organoid cell viability was measured using a CellTiter‐Glo 3D cell viability assay (#G9683; Promega), according to the manufacturer's instructions.

### IHC and Hematoxylin–Eosin Staining and Scoring

Tumor tissues from 447 patients with EOC and patient‐derived organoids were fixed in 10% formalin, embedded in paraffin, and sections. Each section was subjected to IHC staining for SPC25 and MYH9. The stained slides were scanned under a Digital Pathology Slide Scanner (KFBIO, Zhejiang, China), and the results were evaluated by 2 independent pathologists blinded to the clinical outcome. Staining for SPC25 and MYH9 was graded as Negative (no staining positive cells, 0), Weak (0%–30% staining positive cells, +1), Moderate (30%–60% staining positive cells, +2), or Strong (>60% staining positive cells, +3). This staining index was used to assess the protein expression levels in the specimens, and a receiver operating characteristic (ROC) curve was used to determine an optimal threshold to define tumors with high or low expression of the indicated proteins. Specimens exhibiting staining indexes of +3 or +2 were categorized as having high SPC25 or MYH9 expression, whereas those with scores of +1 or 0 were categorized as having low SPC25 or MYH9 expression. Details of the IHC staining results were provided in the Source Data.

### Peptide Synthesis

All synthetic peptides were synthesized by JianDaoShou Biotech (Guangzhou, China). These peptides were purified to a >90% purity via HPLC, suitable for both in vitro and in vivo applications. For in vitro experiments, peptides were dissolved in PBS (Vehicle) to prepare a 10 µM stock solution. For in vivo administration, CBP1 was dissolved in saline (Vehicle) and maintained on ice until injection. Before injection, the solution was equilibrated to room temperature.

### Statistical Analysis

All statistical analyses, including Student's *t*‐test (two‐tailed), chi‐square test, one‐way analysis of variance, and Mantel–Haenszel log‐rank test, were performed using SPSS (version 20.0; IBM Corp., Armonk, NY, USA) and R (version 4.3.3). Kaplan–Meier survival curves were used to determine the differences in survival between 2 or 3 cohorts using the R package “Survminer.” A *p* < 0.05 was considered to indicate statistical significance. Additional information is provided in Supporting Information.

### Ethics Approval

We obtained ethics approval (No. SL‐B2021‐396‐01) from the Institutional Research Ethics Committee of Sun Yat‐sen University Cancer Center and prior patient consent for the use of clinical specimens in this study. All animal experimental procedures were approved by the Institutional Animal Care and Use Committee of Sun Yat‐sen University (No. L102012022228U). Finally, our study complied with the principles of the Declaration of Helsinki.

## Conflict of Interest

The authors declare no conflict of interest.

## Author Contributions

X.J., M.Y., W.Z., and D.S. contributed equally to this work. X.J., M.Y., W.Z., and D.S. performed the majority of the experimental work, including data collection and analysis. W.Z. and Y.L. carried out the qRT‐PCR, western blotting, IF staining, and qRT‐PCR assays. X.J., W.Z., D.S., L.H., S.H., B.C., X.C., and L.K. collected tissues and patient information and conducted IHC and survival analysis. W.Z., M.Y., Y.P., P.D., and R.W. conducted the immunoblotting analysis, plasmid constructions, and IP assays. X.J., S.H., X.C., and Y.L. conducted animal studies. X.J. and Y.O. handled cell culture. D.S., Y.L., and X.L. performed the in vitro studies. Z.L., H.Z., Y.Z., and L.S. conceived the project, designed the experiments, wrote the manuscript, and supervised the study. J.L. provided valuable guidance on the manuscript. The order of the co‐first authors was determined based on their efforts and contributions and efforts to the study.

## Supporting information



Supporting Information

Supporting Information

## Data Availability

The data that support the findings of this study are available from the corresponding author upon reasonable request.
