# Supplementary material for: Targeting the SPC25/RIOK1/MYH9 Axis to Overcome Tumor Stemness and Platinum Resistance in Epithelial Ovarian Cancer
Source: Adv Sci (Weinh). 2024 Nov 3;11(47):2406688. doi: 10.1002/advs.202406688 (PMC11653702; doi:10.1002/advs.202406688)
Supplement: Supplementary file 1 — Supporting Information [file ADVS-11-2406688-s002.docx]

Supporting Information

Targeting the SPC25/RIOK1/MYH9 Axis to Overcome Tumor Stemness and Platinum Resistance in Epithelial Ovarian Cancer

*Xingyu Jiang1 #, Muwen Yang2 #, Weijing Zhang2 #, Dongni Shi1 #, Yue Li1, Lixin He1, Shumei Huang5, Boyu Chen1, Xuwei Chen1, Lingzhi Kong6, Yibing Pan1, Pinwei Deng1, Rui Wang1, Ying Ouyang1, Xiangfu Chen1, Jun Li5, Zheng Li7*, Hequn Zou4*, Yanna Zhang8*, Libing Song1**

**Supplementary Methods**

*Oligonucleotides, Plasmids, Retrovirus Infection, and Stable Cell Line* *Establishment:* cDNAs of human *SPC25*, *RIOK1*, and *MYH9* were cloned into the vector pLVX-retro-hygro to overexpress them in indicated cells or reexpress them in cells with endogenous silencing of the related genes. To silence endogenous *SPC25*, *MYH9*, or *RIOK1*, siRNA targeting the genes was transfected into indicated cells by using Lipofactamine RNAiMax (Life Technologies), or shRNA oligonucleotides were cloned into pSuper-retro-neo or pSuper-retro-puro for the subsequent establishment of stable knockdown cell lines. Target sequences of the oligonucleotides are listed in **Table S4 (Supporting Information)**.

Stable cells were generated from cell pools through retroviral infection by using pLVX-retro-hygro for overexpression of target genes or by using p-Super-retro-neo or pSuper-retro-puro for silencing. In summary, retroviral vectors were cotransfected with the packaging plasmids into HEK293FT cells. The viruses in the supernatants were harvested, and virus infections were induced sequentially over 3 days. Stable cell lines were subsequently established through selection with 0.5 μg/mL puromycin, 25 μg/mL hygromycin, or 250 μg/mL G418.

*Western Blotting:* Western blotting was performed using primary antibodies detailed in **Table S5 (Supporting Information)**. Western blotting grayscale analyses were performed using ImageJ (version 2.0.0; NIH, Bethesda, MD, USA). β-Catenin expression was quantified by determining the gray level of each band using Image J and normalized using α-tubulin.

*Cell Spheroid Formation Assay:* About 1 × 10^3^ cells were plated in six-well ultralow cluster plates and cultured in DMEM/F12 serum-free medium supplemented with 2% B27, 20 ng/mL epidermal growth factor, 20 ng/ml basic fibroblast growth factor, 0.4% bovine serum albumin (BSA), and 5 μg/mL insulin over 10 days. All spheres formed within the wells were visualized under a Zeiss Vert. A1 microscope (Carl Zeiss, Jena, Germany) and spheres with diameters of >50 μm were enumerated using Image-Pro Plus (version 6.0; Media Cybernetics Inc., Rockville, Maryland, USA).

*IF Assay:* About 5 × 10^4^ cells were plated on coverslips, washed three times with PBS, and treated with PBS containing 1% Triton X-100. Next, the cells were blocked in PBS containing 2% BSA at room temperature for 1 h and then incubated with primary antibodies (**Table S5, Supporting Information**) at 4°C overnight, according to the manufacturer’s instructions. The coverslips were subsequently washed using cold PBS and incubated with secondary antibodies (**Table S5, Supporting Information**) diluted with PBS containing 2% BSA at room temperature for 1 h in the dark. Then, the coverslips were counterstained using DAPI (Sigma-Aldrich) to visualize the nuclei. Each sample was observed under an Olympus BX51 microscope (Olympus, Tokyo, Japan). ImageJ (version 2.0.0; NIH, Bethesda, MD, USA) was used to quantify cellular or subcellular fluorescence.

*Colony Formation Assay:* Colony formation assays were conducted to assess the proliferation of indicated cells. Cells (1 × 10^3^) were seeded into six‐well plates and cultured with indicated treatments for 2 weeks. The surviving colonies were fixed with 4% formalin and stained with crystal violet (Beyotime Biotechnology). The total number of colonies was counted using Image-Pro Plus (version 6.0; Media Cybernetics, Rockville, MA, USA).

*TUNEL Assay:* The TUNEL staining procedure was conducted on the DeadEnd Fluorometric TUNEL System (#G3250; Promega), according to the manufacturer’s instructions. In brief, paraffin-embedded sections were baked at 65°C for 60 min, followed by three washes in a xylene bath. The sections were then washed in a series of absolute ethanol, 95% ethanol, and 70% ethanol, and finally, in PBS. Then, each section was treated with proteinase K at 37°C for 30 min and washed three times with PBS. Thereafter, the sections were incubated with equilibration buffer (containing Biotin-11-dUTP and TdT Enzyme) at 37 ℃ for 1 h in the dark, followed by three PBS washes. The samples were subsequently incubated with streptavidin–fluorescein at 37 ℃ for 30 min in the dark, followed by three washes with PBS. DAPI was used to stain the nucleus. Finally, the experimental outcomes were observed under a fluorescence microscope, and the percentages of TUNEL-positive cells were quantified from 10 randomly selected fields.

*Cell Viability Assay:* The indicated cells were seeded into 96-well plates at a density of 4 × 10^3^ cells per well. Next, the Cell Counting Kit-8 reagent (Dojindo) was added to the wells, followed by incubation at 37°C for 2 h in the dark, after which the optical density at 450 nm was determined on a microplate reader (EPOCH2; BioTek, USA). Each cell line was represented using triplicate wells, and the experiment was repeated three times.

*Luciferase Reporter Assay:* The indicated cells (3 × 10^3^) were cultured in triplicate in 48‐well plates for 24 h. Subsequently, the cells transfected with 100 ng of luciferase reporter plasmid or control luciferase plasmid, along with 1 ng of pRL‐TK Renilla plasmid (Promega), by utilizing Lipofectamine 3000 (Invitrogen), according to the manufacturer’s protocol. The Renilla luciferase signals were assessed 24 h after transfection using a Dual-Luciferase Reporter Assay Kit (Promega), according to the manufacturer’s protocol.

*Fluorescence-Activated Cell Sorting Assay:* For apoptosis analysis, we used an Annexin V-FITC/PI Apoptosis Detection Kit (#A211-01; Vazyme Biotech), according to the manufacturer’s instructions. We considered the total proportion of Annexin V-positive cells (quadrants II and III) to be the apoptotic rate, regardless of the PI status. All samples were analyzed on a flow cytometer (cytoFLEX LX; Beckman Coulter, Indianapolis, IN, USA), and percentages of apoptotic cells were analyzed using CytExpert (Beckman Coulter).

For CD133-positive cell subpopulation analysis, the indicated cells were digested with trypsin and suspended in Hank’s balanced salt solution containing 2% FBS. Subsequently, 5 × 10^5^ cells were incubated with negative control antibodies, isotype control antibodies (#554680; BD, Bedford, MA, USA), or anti-CD133 (#566593; BD) at 4°C for 30 min. The cells were then washed to remove unbound antibodies and analyzed on the flow cytometer (cytoFLEX LX). The percentages of CD133-positive cells were analyzed using FlowJo (Tree Star, Ashland, OR, USA).

*RIP Assay:* We performed RIP assays to ascertain the interactions between eIF4E and *CTNNB1* mRNA in EOC cells. To initiate robust gene transcription, the cells were subjected to a 24-h period of starvation, followed by stimulation with a standard culture medium supplemented with 10% FBS. Subsequently, cell lysates were prepared using lysis buffer (comprising 20 mmol/L Tris-Cl, pH = 8.0, 10 mmol/L NaCl, 1 mmol/L EDTA, and 0.5% NP-40) supplemented with RNasin (#N2111S; Promega). These lysates were then incubated with antibodies targeting eIF4E (#2067; Cell Signaling Technology) or immunoglobulin G (#I8765; Sigma‐Aldrich) and then washed five times with lysis buffer. The resulting pellets were subjected to qRT-PCR with primers specific to *CTNNB1*. *GAPDH* was used the negative control. **Table S3 (Supporting Information)** lists the primers used here.

*ChIP Assay:* About 4 × 10^6^ of the indicated cells were treated with 1% (final concentration) formaldehyde to facilitate the crosslinking of proteins to DNA. The reaction was terminated by the addition of glycine (#G8790; Sigma-Aldrich). Subsequently, the cell lysates were subjected to sonication to generate DNA fragments 300–1,000 base pairs in length. The resulting chromatin supernatants were then subjected to overnight incubation at 4°C with rotation in the presence of specific antibodies targeting RNAPII (#05‐623; Millipore) or immunoglobulin G. After the reversal of the crosslinking in protein–DNA complexes to release DNA, qRT-PCR amplification was conducted using primers specific to *CTNNB1*. *GAPDH* was used as the negative control. **Table S3 (Supporting Information)** lists the primers used here.

*TOP/FOP Flash Assay:* The wild-type (TOP) and mutant (FOP) LEF/TCF promoter regions were engineered into pGL3 luciferase constructs (Promega). The indicated cells (at 2 × 10^4^ per well) were distributed in triplicate across 48-well plates and allowed to settle for 24 h. Next, 100 ng of TOP-Flash or FOP Flash and 1 ng of pRL-TK Renilla plasmid (Promega) were transfected into the cells using Lipofectamine 3000 (#L3000015; Thermo Fisher), according to the manufacturer's instructions. Renilla luciferase signals were assessed 24 h after transfection by using a Dual-Luciferase Reporter Assay Kit (#E1910; Promega), according to the manufacturer’s protocol. The outcome was determined as the ratio of specific TOP-Flash to nonspecific FOP Flash relative to the Renilla luciferase units.

*LC-MS Analysis:* To identify proteins potentially interacting with SPC25 or MYH9, OVCAR8 cells were transfected with Flag-SPC25 or Myc-MYH9. Cytoplasmic lysates were extracted and then immunoprecipitated with Flag-beads (#A2220; Millipore) or Myc-beads (#9B11; Cell Signaling Technology). The beads containing affinity-bound proteins were washed six times using a wash buffer (150 mM NaCl, 10 mM HEPES, 0.1% NP-40, pH = 7.4), followed by elution using 1 M glycine (pH = 3.0). Elutes were subjected to MS, and the full list of the SPC25- and MYH9-interacting proteins is provided in **SourceData**.

*Nuclear and Cytoplasmic Extract Isolation:* Nuclear and cytoplasmic extracts were prepared using an NE-PER Nuclear Cytoplasmic Extraction Reagent kit (Pierce, Rockford, IL, USA), according to the manufacturer’s protocol. First, OVCAR8 cells were washed twice with PBS and centrifuged at 500 × g for 5 min. Next, the cell pellets were suspended in 200 μL of cytoplasmic extraction reagent I, mixed through vortexing, and subsequently incubated on ice for 10 min. Subsequently, 11 μL of cytoplasmic extraction reagent II was added, followed by vortexing for 5 s, incubation on ice for 1 min, and centrifugation at 16,000 × g for 5 min. The supernatant constituting the cytoplasmic extract was used for further experimentation. The insoluble pellet fraction, containing crude nuclei, was resuspended in 100 μL of nuclear extraction reagent through vortexing for 15 s, incubated on ice for 10 min, and centrifuged at 16,000 × g for 10 min. The resulting supernatant including the nuclear extract was used for subsequent experimentation.

*Co-IP Assay:* Cell lysates were generated from the indicated cells by using lysis buffer (150 mM NaCl, 10 mM HEPES, 0.1% NP-40, pH = 7.4). These lysates were subsequently subjected to overnight incubation at 4°C with indicated antibodies or conjugated beads (**Table S5, Supporting Information**). Thereafter, beads containing proteins bound to the antibodies were thoroughly washed six times with an IP wash buffer (150 mM NaCl, 10 mM HEPES, 0.1% NP-40, pH = 7.4), followed by elution using 1 M glycine at pH 3.0. The resulting elutes were mixed with the sample buffer, denatured, separated through SDS-PAGE, and used for subsequent Western blotting analysis.

*PLA:* Proximity ligation was conducted using a Rabbit PLUS and Mouse MINUS Duolink in situ PLA kit (Sigma-Aldrich), according to the manufacturer’s instructions. First, OVCAR8 cells were seeded onto coverslips, fixed in 3.7% formaldehyde in PBS at room temperature for 15 min, and subsequently washed with tris-buffered saline (TBS). Next, the cells were blocked for 2 h with 1% BSA in TBST (TBS with 1‰ Tween-20) in a humidified chamber at room temperature. The coverslips were then incubated with anti-SPC25 and anti-RIOK1 (**Table S5, Supporting Information**) at 4°C overnight. After a wash with TBST, proximity ligation was executed using a PLA kit (Sigma-Aldrich), and the cells were counterstained with DAPI (Sigma-Aldrich) to visualize nuclei. PLA signals were observed under an Olympus BX51 microscope (Olympus, Tokyo, Japan) with 40× magnification and analyzed a macro of ImageJ (version 2.0.0). PLA signals were quantified by tallying foci per cell from five random fields.

*Analysis of Surface Plasmon Resonance:* Analysis of Surface Plasmon Resonance Binding kinetics between SPC25 and the indicated peptide-CBP1 were measured by Surface Plasmon Resonance (SPR) using a BIAcore T200 instrument (GE Healthcare, CA, USA). The dissociation constant (Kd) was calculated according to the BIA-evaluation software.

*Gene Expression Profiling and Analysis:* Gene expression profiling and analysis were performed based on the public data from TCGA (https://portal.gdc.cancer.gov/) and Gene Expression Omnibus data sets (GSE148003, GSE141630, GSE149146, and GSE198042). The mRNA stemness index data were obtained from previous research work of Tathiane et al ^[1]^. GSEA was performed using the R package ‘ClusterProfile.’

**Supplementary Tables**

**Table S1.** Clinicopathological characteristics of 447 patients with EOC.

| **Characteristic** | **No. (%) of cases** |
| --- | --- |
| **Age (years)**  <54 | 239 (53.5) |
| ≥54 | 208 (46.5) |
| **FIGO stage** |  |
| I-II | 132 (29.5) |
| III-IV | 315 (70.5) |
| **Histologic grade**  G1-2  G3  **Pathology**  Serous carcinoma  Mucinous carcinoma  Endometrioid carcinoma  Clear cell carcinoma  Other  **Vital status**  Alive  Dead  **Platinum type**  Cisplatinum  Carboplatinum  **Therapy response**  CR/PR  SD/PD  **SPC25 staining level**  Low  High  **Nuclear MYH9 staining level**  Low  High | 179 (40.0)  268 (60.0)  274 (61.3)  76 (17.0)  57 (12.8)  34 (7.6)  6 (1.3)  314 (70.2)  133 (29.8)  104 (23.3)  344 (76.7)  333 (74.5)  114 (25.5)  245 (54.8)  202 (45.2)  223 (49.9)  224 (40.1) |

**Table S2**. Correlation between SPC25 and clinicopathological characteristics.

| **Characteristic** | **SPC25 staining level** | | ***P*** |
| --- | --- | --- | --- |
|  | **Low**  **(No. of cases)** | **High**  **(No. of cases)** |  |
| **Age (years)**  <54  ≥54 | 135  110 | 104  98 | 0.506 |
| **FIGO stage**  I-II  III-IV | 76  169 | 56  146 | 0.512 |
| **Histological grade**  G1-2  G3 | 90  155 | 89  113 | 0.14 |
| **Pathology**  Serous carcinoma  Mucinous carcinoma  Endometrioid carcinoma  Clear cell carcinoma  Other | 156  47  22  17  3 | 118  29  35  17  3 | 0.763 |
| **Platinum type**  Cisplatinum  Carboplatinum  **Therapy response**  CR/PR  SD/PD  **Nuclear MYH9 staining level**  Low  High | 60  185  200  45  141  104 | 44  158  133  69  82  120 | 0.574  < 0.0001  < 0.0001 |

**Table S3.** PCR primers used in this study.

| **Gene** | **Sequence (5′-3′)** |
| --- | --- |
| *SPC25* | Forward: AGTACGGACACCTCCTGTCAG  Reverse: TCTCAACCATTCGTTCTTCTTCC |
| *OCT4* | Forward: CCTGAAGCAGAAGAGGATCACC  Reverse: AAAGCGGCAGATGGTCGTTTGG |
| *SOX2* | Forward: GCCGAGTGGAAACTTTTGTCG  Reverse: GGCAGCGTGTACTTATCCTTCT |
| *NANOG* | Forward: TTTGTGGGCCTGAAGAAAACT  Reverse: AGGGCTGTCCTGAATAAGCAG |
| *C-MYC* | Forward: TGAGGAGGAACAAGAAGATG  Reverse: ATCCAGACTCTGACCTTTT |
| *C-JUN* | Forward: TCCAAGTGCCGAAAAAGGAAG  Reverse: CGAGTTCTGAGCTTTCAAGGT |
| *KLF4*  *AXIN2*  *SURVIVIN*  *TCF-1*  *CTNNB1*  *CTNNB1* (for ChIP assay)  *MYH9*  *RIOK1*  *GAPDH*  *GAPDH* (for ChIP assay) | Forward: AGCTTCTTTAATCCCGTCTGTG  Reverse: GGCCAGAGCCCGTTTCTTT  Forward: ACAACAGCATTGTCTCCAAGCAGC  Reverse: GCGCCTGGTCAAACATGATGGAAT  Forward: CATCTCTACATTCAAGAACTGG  Reverse: CCTTGAAGCAGAAGAAACAC  Forward: AGACTATGCTCATCACCG  Reverse: GTCTGAGGTGAAGACCTG  Forward: GCTTGTTCGTGCACATCAGGATA  Reverse: GGCTCCGGTACAACCTTCAACTA  Forward: CGGGGTACCTCAGACGGCAGCAGACT  Reverse: CCCAAGCTTGAGAGGCTTAAAATGGCG  Forward: CCTCAAGGAGCGTTACTACTCA  Reverse: CTGTAGGCGGTGTCTGTGAT  Forward: GGCTCGGGAGTTGTACCTG  Reverse: CCACGGACTGAGACACGTC  Forward: ATGGGGAAGGTGAAGGTCG  Reverse: GGGGTCATTGATGGCAACAATA  Forward: CTGAGCAGACCGGTGTCACATC  Reverse: GAGGACTTTGGGAACGACTGAG |

**Table S4.** Sequences of oligonucleotides and shRNAs used in this study for gene silencing.

| **Target** | **Target sequence (5′-3′)** |
| --- | --- |
| sh-*SPC25*#1/si-*SPC25*#1 | GAATTTCAAGAGAATGTAA |
| sh-*SPC25*#2/si-*SPC25*#2 | GGACTAAGAGATACCTACA |
| si-*CTNNB1* | GGGTAGGGTAAATCAGTAA |
| sh-*MYH9*#1 | CCGTACAACAAATACCGCT |
| sh-*MYH9*#2 | GGGTATCAATGTGACCGAT |
| sh-*RIOK1*#1 | GGAGGCGTGTATATCATTG |
| sh-*RIOK1*#2 | GAGCATATATTCCTAGAAC |

**Table S5.** Antibodies and conjugated beads used in this study.

| **Catalog No.** | **Antibodies** | **Species** | **Company** | **Application** |
| --- | --- | --- | --- | --- |
| #HPA047144 | Anti-SPC25 | Rabbit | Sigma-Aldrich | IHC/WB |
| #ab242101 | Anti-SPC25 | Rabbit | Abcam | Co-IP |
| #2125 | Anti-α-Tubulin | Rabbit | Cell Signaling Technology | WB |
| #ab110315 | Anti-Cleaved-PAPR1 | Mouse | Abcam | WB |
| #66470-1-Ig | Anti-Caspase-3 | Mouse | Proteintech Group | WB |
| #8814 | Anti-β-Catenin (Active) | Rabbit | Cell Signaling Technology | WB |
| #sc-7199 | Anti-β-Catenin | Rabbit | Santa Cruz Biotechnology | WB/IF |
| #2750 | Anti-OCT4 | Rabbit | Cell Signaling Technology | WB |
| #3580 | Anti-Nanog | Rabbit | Cell Signaling Technology | WB |
| #5605 | Anti-c-Myc | Rabbit | Cell Signaling Technology | WB |
| #9165 | Anti-c-Jun | Rabbit | Cell Signaling Technology | WB |
| #11880-1-AP | Anti-KLF4 | Rabbit | Proteintech Group | WB |
| #ab109307 | Anti-AXIN2 | Rabbit | Abcam | WB |
| #2808 | Anti-Survivin | Rabbit | Cell Signaling Technology | WB |
| #2203 | Anti-TCF1 | Rabbit | Cell Signaling Technology | WB |
| #sc-98978 | Anti-MYH9 | Rabbit | Santa Cruz Biotechnology | WB/Co-IP |
| #5026 | Anti-Phospho-Myosin IIa (Ser1943) | Rabbit | Cell Signaling Technology | WB |
| #14793 | Anti-Flag | Rabbit | Cell Signaling Technology | WB/Co-IP |
| #16286-1-AP | Anti-Myc | Rabbit | Proteintech Group | WB/Co-IP |
| #AP1067 | Anti-pan-Phospho-Ser/Thr | Mouse | ABclonal | WB |
| #ab16048 | Anti-Lamin B1 | Rabbit | Abcam | WB |
| #17222-1-AP | Anti-RIOK1 | Rabbit | Proteintech Group | WB/Co-IP |
| #51064-2-AP | Anti-HA | Rabbit | Proteintech Group | WB/Co-IP |
| #2622 | Anti-GST | Rabbit | Cell Signaling Technology | WB |
| #A2220 | Anti-Flag M2 Affinity Gel | Mouse | Millipore | Co-IP |
| #9B11 | Anti-Myc (Sepharose^®^ Bead Conjugate) | Mouse | Cell Signaling Technology | Co-IP |
| #SAE0197 | Anti-HA Magnetic Beads | Mouse | Sigma-Aldrich | Co-IP |
| #ab7090 | Antirabbit immunoglobulin G | Goat | Abcam | Co-IP |
| #P2173 | IgG Magnetic Beads | Rabbit | Beyotime | Co-IP |
| #P2171 | IgG Magnetic Beads | Mouse | Beyotime | Co-IP |
| #A11008 | Alexa Fluor 488 labeled antirabbit | Goat | Life Technologies | IF |
| #566593 | PE-Anti-CD133 | Mouse | BD | FACs |

**Table S6.** Chemicals and recombinant proteins used in this study.

| **Chemical or recombinant protein** | **Catalog No.** | **Manufacturer** |
| --- | --- | --- |
| Cisplatinum | #HY-17394 | MedChem Express |
| XAV939 | #X3004 | Sigma-Aldrich |
| Wnt-3a | #5036-WN | R&D |
| CHX | #239765 | Sigma-Aldrich |
| Act D | #A4262 | Sigma-Aldrich |
| IPTG | #1140GR005 | BioFroxx |
| CIAP | #18009019 | Invitrogen |
| DSS | #S0657 | Selleckchem |
| CX-4945 | # HY-50855 | MedChem Express |

**Supplementary Figures and Legends**

**
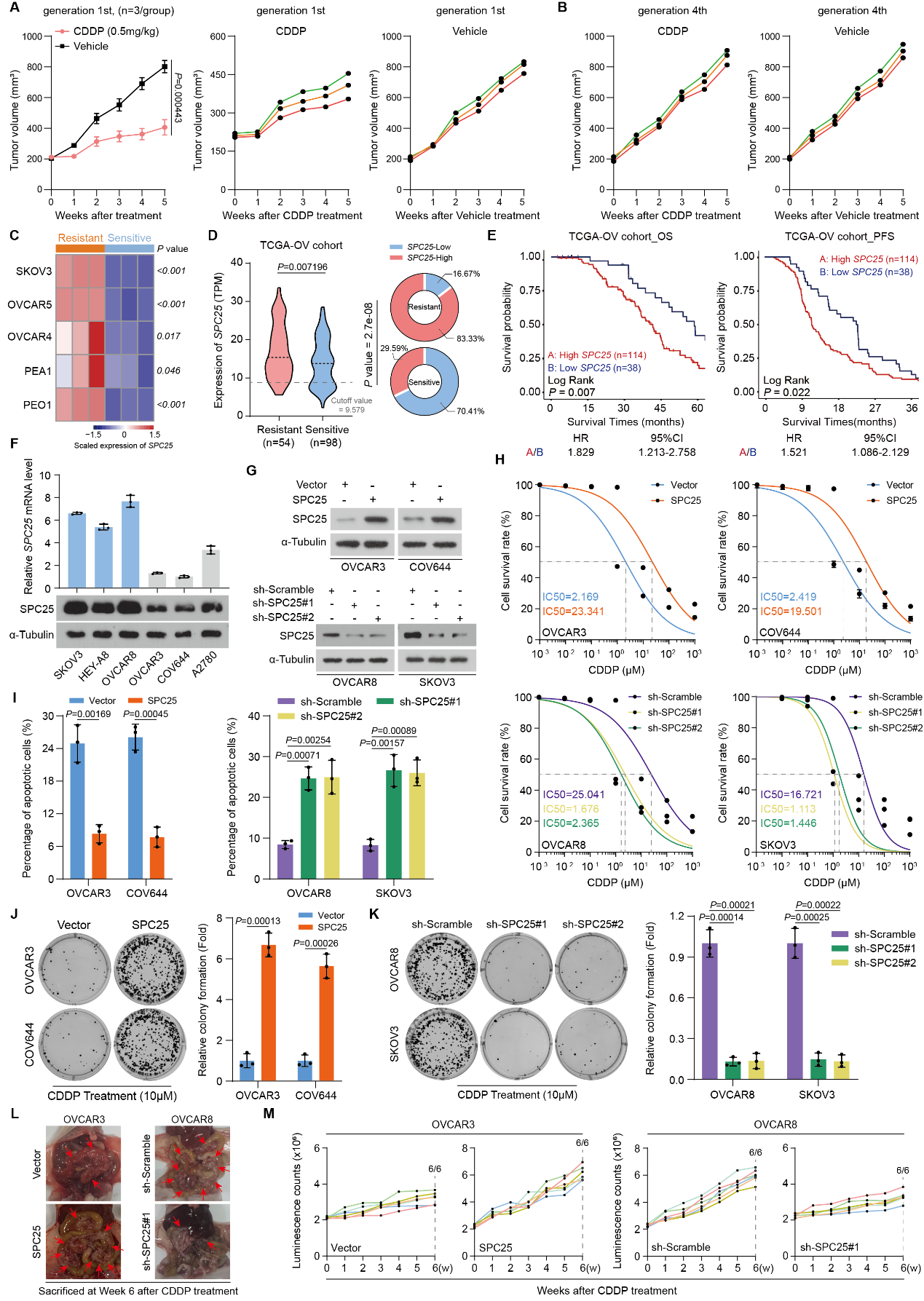
**

**Figure S1.** A,B) Curves of first-generation (A) and fourth-generation (B) xenografted tumor volumes calculated in the indicated weeks (*n* = 3 per group). C) Heatmap of *SPC25* expression across transcriptome datasets from various platinum-resistant EOC cell lines. D) *SPC25* expression in platinum-resistant and -sensitive groups in the TCGA-OV cohort (left). Correlation of *SPC25* expression (cutoff = 9.579) with response to platinum-based adjuvant chemotherapy (right) based on the chi-square test. E) Kaplan–Meier survival analysis of TCGA-OV cases stratified by low and high *SPC25* expression. Mantel–Haenszel log-rank test was used. F) Endogenous expression of SPC25 in six human EOC cell lines detected through qRT-PCR and Western blotting. G) Exogenous *SPC25* overexpression (upper) and silencing (lower) in OVCAR3 and COV644 cells. H) Cell Counting Kit-8 assay results showing that cell viability was significantly enhanced in *SPC25*-overexpressing OVCAR3 and COV644 cells after CDDP treatment (upper) but significantly impaired in *SPC25*-silenced OVCAR8 and SKOV3 cells (lower). I) Quantification of fluorescence-activated cell sorting analysis of annexin V/PI staining in OVCAR3, COV644, OVCAR8, and SKOV3 cells under CDDP (10 μM) treatment. J) Representative images of surviving colonies of *SPC25*-overexpressing OVCAR3 and COV644 cells under CDDP (10 μM) treatment (left). Quantification of surviving colonies (right). K) Representative images of surviving colonies of *SPC25*-silenced OVCAR8 and SKOV3 cells under CDDP (10 μM) treatment (left). Quantification of survived colonies (right). L) Representative images of intraperitoneal tumor-bearing nude mice sacrificed on week 6 after CDDP treatment. M) Relative changes in luminescence signal of intraperitoneal tumors in nude mice receiving CDDP treatment in the indicated weeks (*n* = 6 per group). In (H), (I), (J), and (K), *n* = 3 biological replicates. Error bars represent the means ± standard deviations of independent experiments. In (D), (I), (J), and (K), two-sided Student’s *t* test was used. In (A) and (H), one-way repeated-measures analysis of variance was used.


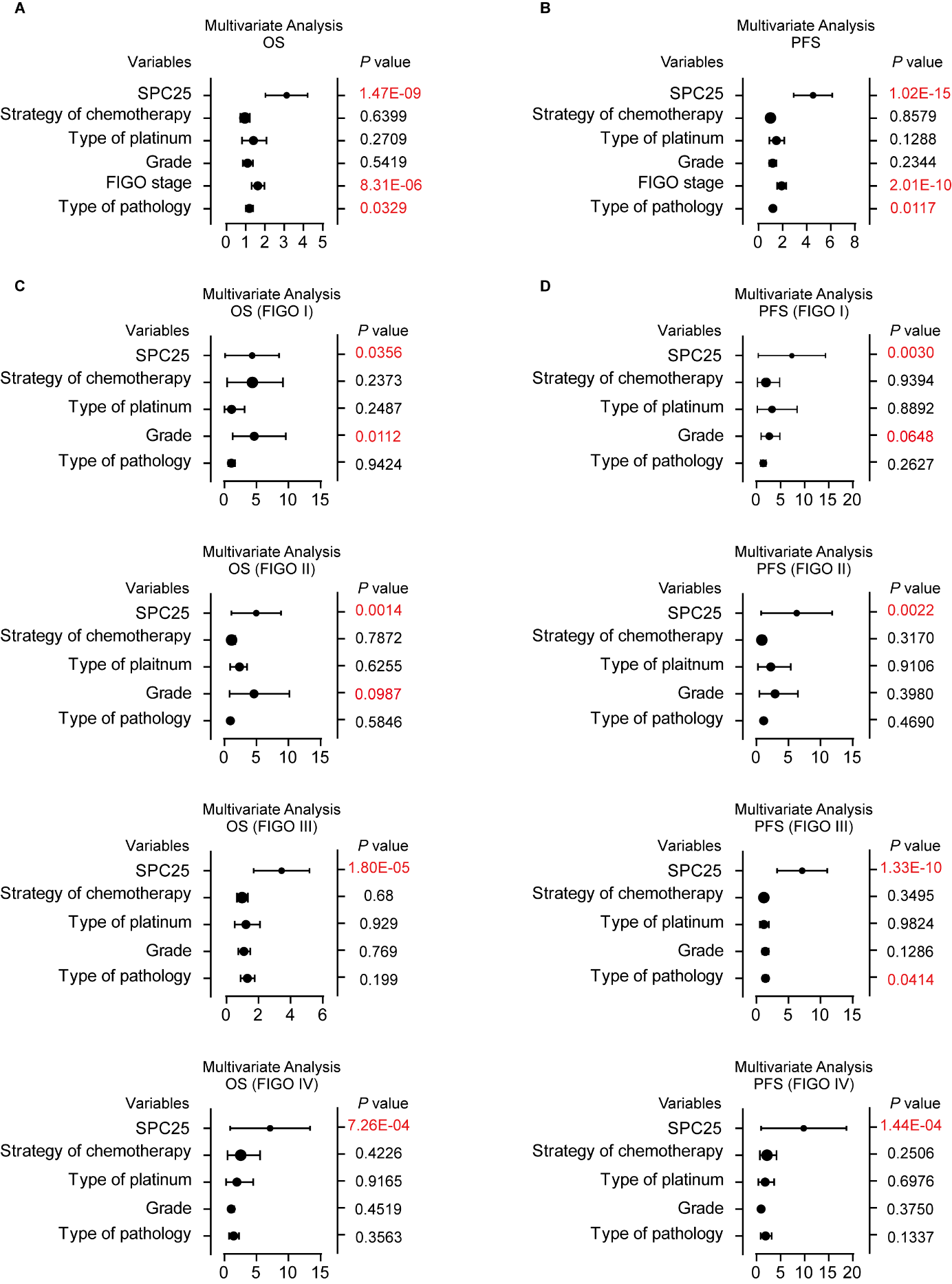


**Figure S2.** A) Forest plot of the hazard ratio (HR) for the association of SPC25 expression with overall survival. B) Forest plot of the hazard ratio (HR) for the association of SPC25 expression with progression-free survival. C) Forest plot of the hazard ratio (HR) for the association of SPC25 expression with overall survival in different FIGO stage. D) Forest plot of the hazard ratio (HR) for the association of SPC25 expression with progression-free survival in different FIGO stage.


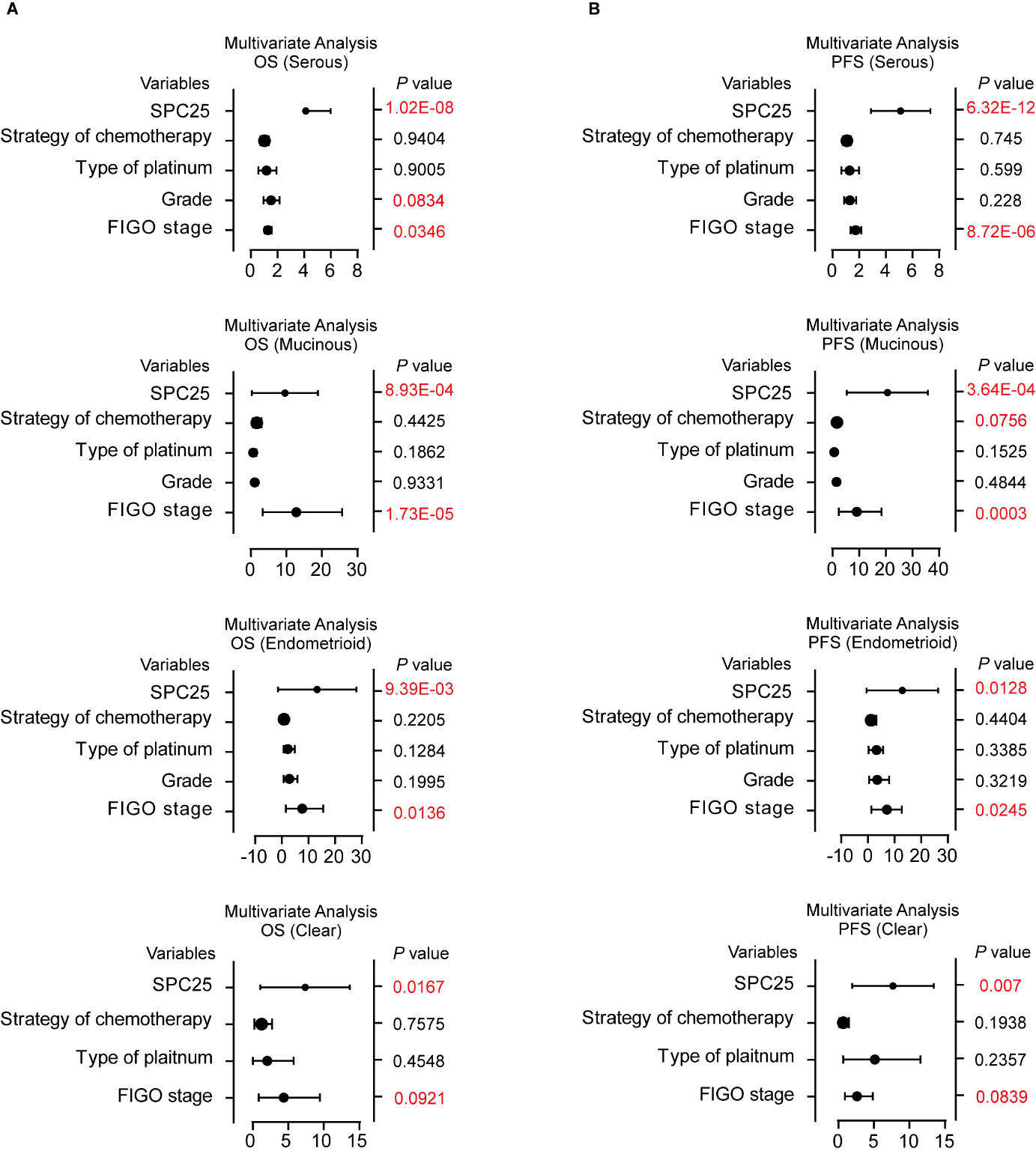

**Figure S3.** A) Forest plot of the hazard ratio (HR) for the association of SPC25 expression with overall survival in different pathological subtypes. B) Forest plot of the hazard ratio (HR) for the association of SPC25 expression with progression-free survival in different pathological subtypes.

**
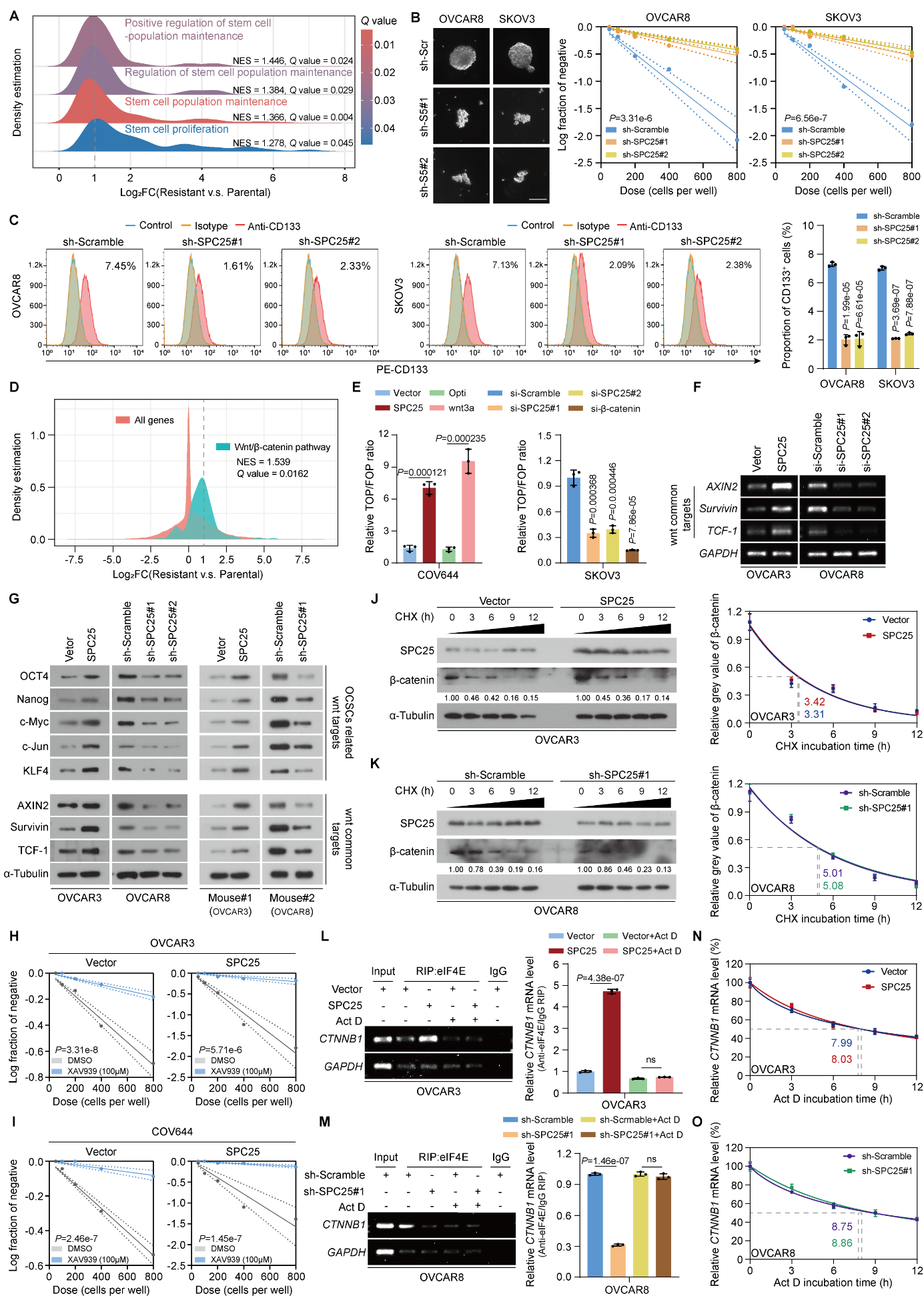
**

**Figure S4.** A) Density plot demonstrating the GSEA results. B) Representative images of tumor-spheres formation the limitation dilution assays of OVCAR8 and SKOV3 cells with *SPC25*-silenced (left). Log-dose slope of the scramble and SPC25-silenced groups (right). Scale bar, 200 μm. C) Flow cytometry assays of CD133-positive subpopulations of OVCAR8 and SKOV3 cells. D) Density plot demonstrating the GSEA results. E) Relative ratio of luciferase activities of TOP-Flash over FOP-Flash normalized using Renilla luciferase activity in COV644 and SKOV3 cells after the indicated treatment (Wnt-3a, 1.6 ng/mL, overnight). F) qRT-PCR for the common Wnt target genes *AXIN2*, the Survivin gene, and *TCF1* in control, *SPC25*-overexpressing, and *SPC25*-silenced EOC cells. G) Expression levels of total Wnt target genes in EOC cell lines and xenografted tumors after indicated treatments assessed through Western blotting. H) Representative images of tumor sphere formation assays of *SPC25*-overexpressing OVCAR3 cells after XAV939 treatment (left). Quantification of formed tumor spheres (right). I) Representative images of tumor sphere formation assays of *SPC25*-overexpressing COV644 cells after XAV939 treatment (left). Quantification of formed tumor spheres (right). J) Expression of β-catenin in control or *SPC25*-overexpressing OVCAR3 cells after treatment with CHX (0.1 mg/mL) for the indicated time assessed through Western blotting (right). Graph showing the level of β-catenin remaining after CHX treatment as a percentage of the starting β-catenin level (left). K) Expression of β-catenin in control or *SPC25*-silenced OVCAR8 cells after treatment with CHX (0.1 mg/mL) for the indicated time assessed through western blotting (right). Graph showing the level of β-catenin remaining after CHX treatment as a percentage of the starting β-catenin level (left). L,M) Interactions between eIF4E and the indicated mRNAs in control or *SPC25*-overexpressing (L) and *SPC25*-silenced (M) OVCAR3 cells with or without Act D treatment examined using RIP assays. N,O) qRT-PCR analysis of β-catenin mRNAs after Act D treatment on the indicated time in control or *SPC25*-overexpressing (N) and *SPC25*-silenced (O) OVCAR8 cells. In (C), (E), (J), (K), (L), (M), (N) and (O), *n* = 3 biological replicates. Error bars represent the means ± standard deviations of independent experiments; ns denotes not significant. In (B), (C), (E), (H), (I), (L), and (M), two-sided Student’s *t* test was used. In (J), (K), (N), and (O), one-way repeated-measures analysis of variance was used.

**
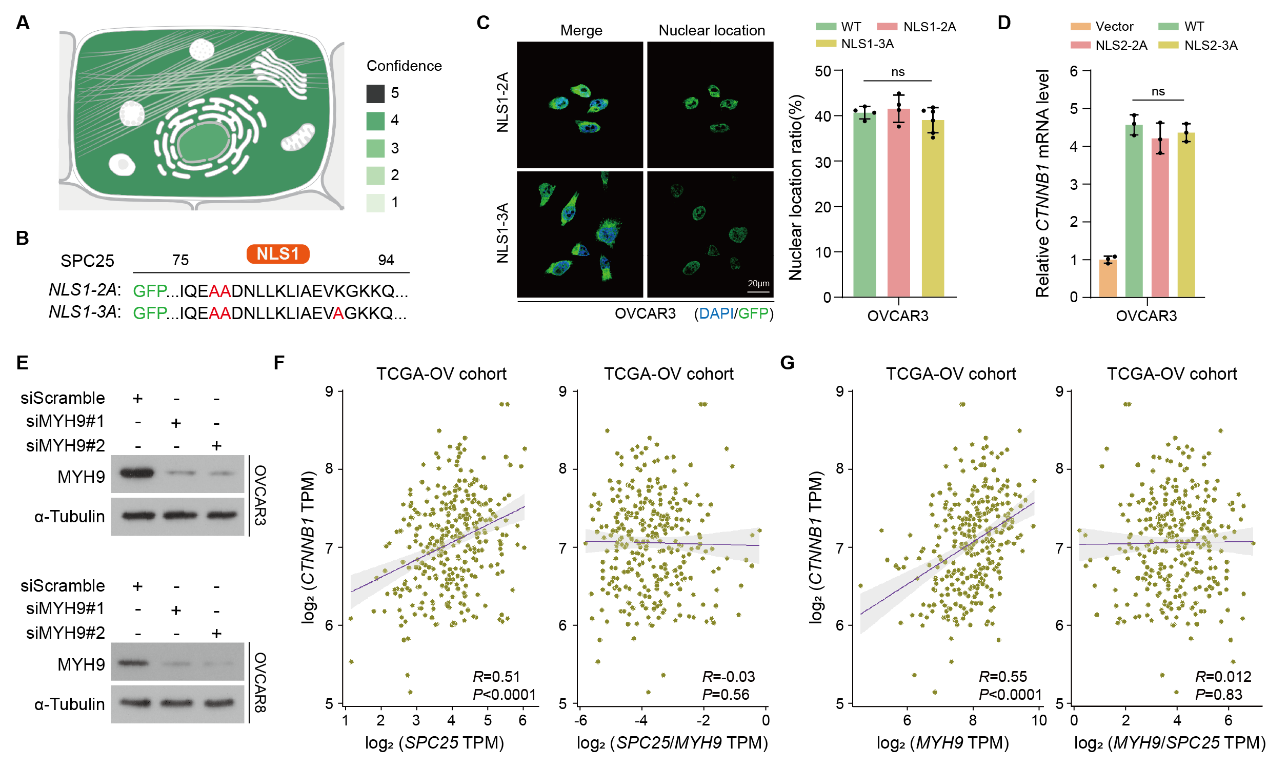
**

**Figure S5.** A) Schematic demonstrating confidence for subcellular locations of SPC25 based on previous studies. B) Schematic of NLS1 sequences of NLS1-2A (two-point mutations) and NLS1-3A (three-point mutations). Red letters indicate critical sites in SPC25 NLS1. C) Fluorescence of expressed GFP-SPC25 with NLS1-2A and NLS1-3A mutants in OVCAR3 cells (left). Quantitation of the ratio of the nuclear GFP luminescence (right; 4–10 GFP-positive cells counted under 60× field of view). Scale bar, 20 μm. D) qRT-PCR of *CTNNB1* expression in OVCAR3 cells transfected with WT, NLS2-2A, and NLS2-3A mutants of *SPC25*. E) Endogenous silencing of *MYH9* in OVCAR3 and OVCAR8 cells. F) Correlation of *CTNNB1* expression with *SPC25* expression or *MYH9*-corrected *SPC25* expression based on TCGA-OV data. Spearman correlation analysis was used here. G) Correlation of *CTNNB1* expression with *MYH9* expression or *SPC25*-corrected *MYH9* expression based on TCGA-OV data. Spearman correlation analysis was used here. Error bars represent the means ± standard deviations of independent experiments; ns denotes not significant. In (C) and (D), one-way analysis of variance was used.

**
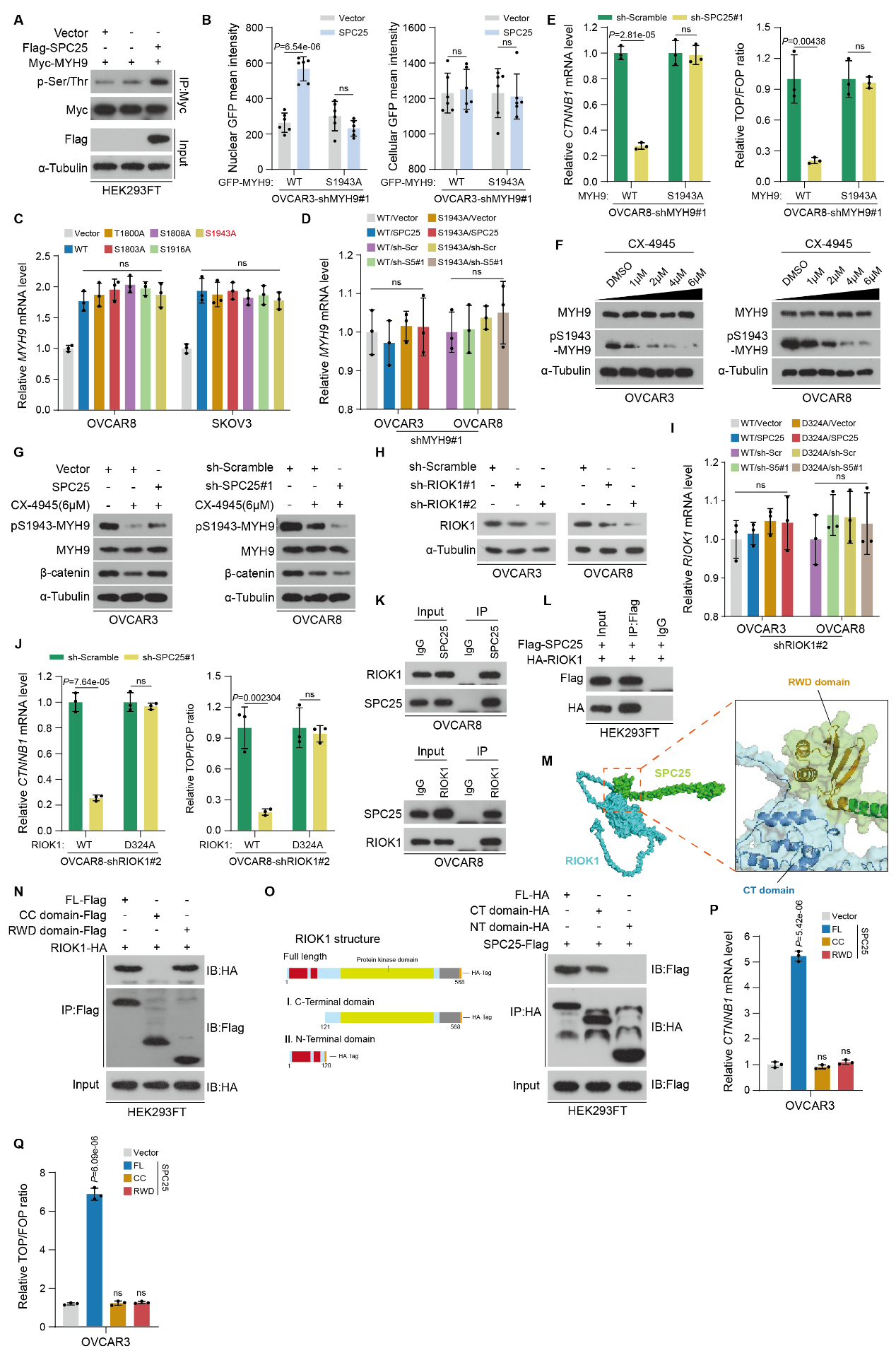
**

**Figure S6.** A) Exogenous IP assays performed to assess Ser/Thr phosphorylation status of MYH9-Myc in HEK293FT cells with or without *SPC25-Flag* expression. B) Quantification of nuclear or cellular fluorescence of reexpressed GFP-MYH9-WT with NLS2-2A and NLS2-3A mutants in *MYH9*-silenced OVCAR3 cells with or without SPC25 overexpression. C) qRT-PCR of *MYH9* in OVCAR8 and SKOV3 cells with exogenous expression of MYH9 mutant variants. D) qRT-PCR of *MYH9* in *MYH9*-silenced OVCAR3 and OVCAR8 cells with reexpression of *MYH9*-WT or *MYH9*-S1943A. E) qRT-PCR of *CTNNB1* in *MYH9*-silenced OVCAR8 cells with reexpression of *MYH9*-WT or *MYH9*-S1943A under *SPC25* silencing (left). TOP/FOP Flash analysis in *MYH9*-silenced OVCAR8 cells with reexpression of *MYH9*-WT or *MYH9*-S1943A under *SPC25* silencing (right). F) Endogenous phosphorylation status of MYH9 at Ser1943 in OVCAR3 and OVCAR8 cells under escalating doses of CX-4945 examined through Western blotting. G) Phosphorylation status of MYH9 at Ser1943 and β-catenin levels in OVCAR3 and OVCAR8 cells with *SPC25* overexpression or silencing under the treatment of CX-4945 (6 μM) examined through Western blotting. H) Endogenous silencing of *RIOK1* in OVCAR3 and OVCAR8 cells. I) qRT-PCR of *RIOK1* in *RIOK1*-silenced OVCAR8 cells with reexpression of *RIOK1*-WT or *RIOK1*-D324A. J) qRT-PCR of *CTNNB1* in *RIOK1*-silenced OVCAR8 cells with reexpression of *RIOK1*-WT or *RIOK1*-D324A under *SPC25* silencing (left). TOP/FOP Flash analysis in *RIOK1*-silenced OVCAR8 cells with reexpression of *RIOK1*-WT or *RIOK1*-D324A under *SPC25* silencing. K,L) Endogenous interaction between SPC25 and RIOK1 validated using IP assays in OVCAR8 cells. L) Exogenous interaction between SPC25 and RIOK1 validated using IP assays in HEK293FT cells transfected with SPC25-Flag and RIOK1-HA constructs. M) Structural model of protein complex composed of SPC25 and RIOK1 monomers predicted using AlphaFold2. N) HEK293FT cells transfected with indicated RIOK1-HA and SPC25-Flag truncations, followed by IP assays with Flag-beads, for assessing their interactions with RIOK1 precisely. O) Schematic of RIOK1 protein truncated constructs (left). HEK293FT cells were transfected with indicated SPC25-Flag and RIOK1-HA truncations, followed by IP assays with HA-beads, for assessing their detailed interactions with SPC25 precisely (right). P) qRT-PCR of *CTNNB1* in OVCAR3 cells with exogenous expression of FL or truncated Flag-tagged SPC25 constructs. Q) TOP/FOP Flash analysis in OVCAR3 cells with exogenous expression of FL or truncated Flag-tagged SPC25 constructs. In (B), (E), (C), (D), (I), (J), (P) and (Q), *n* = 3 biological replicates. Error bars represent the means ± standard deviations from independent experiments; ns denotes not significant. In (B), (E), (J), (P), and (Q), two-sided Student’s *t* test was used. In (C), (D), and (I), one-way analysis of variance was used.

**
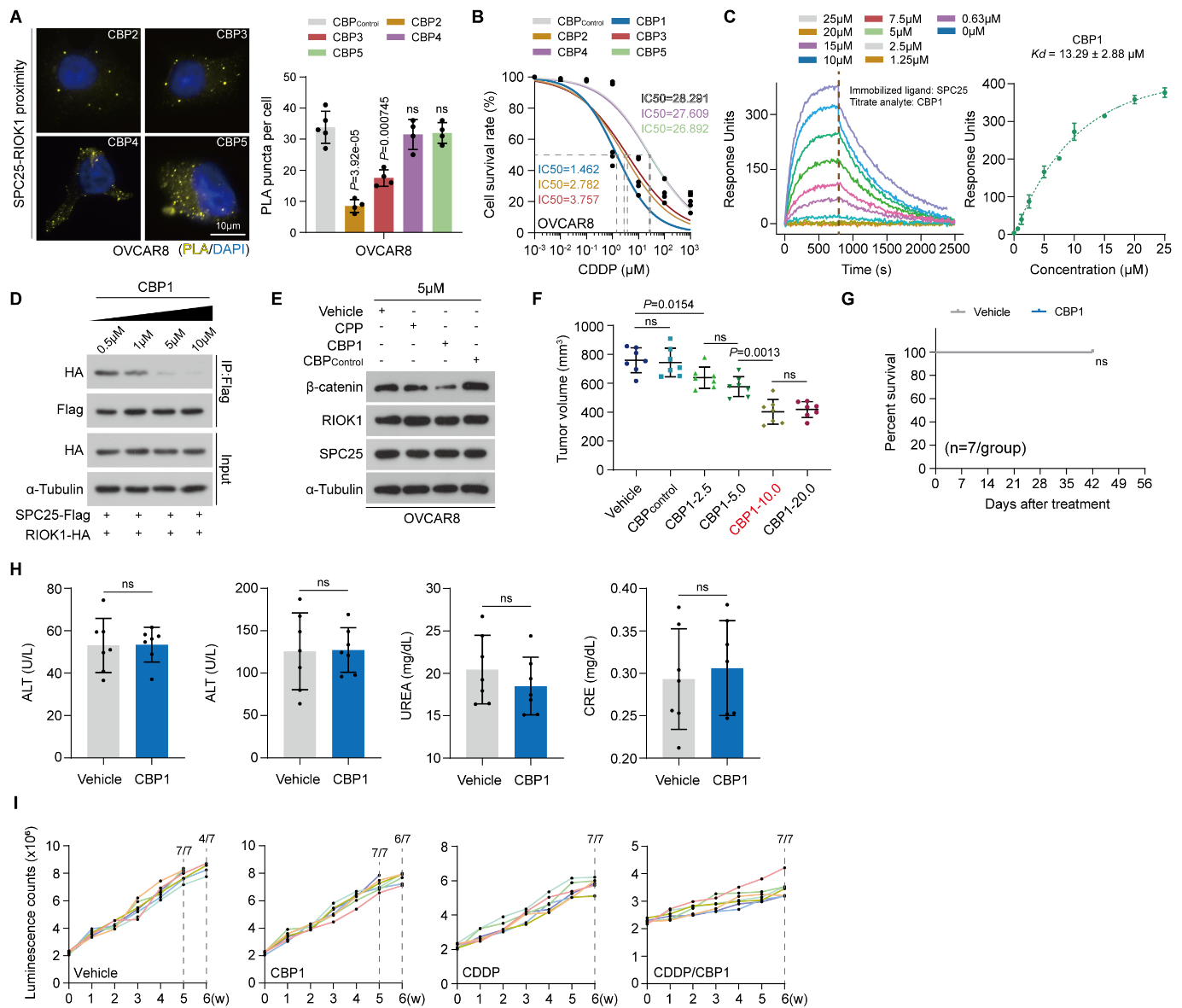
**

**Figure S7.** A) Representative images and quantification of PLA signals indicating SPC25–RIOK1 interaction in OVCAR8 cells treated with CBP2, CBP3, CBP4, or CBP5. PLA signals were quantified by counting the foci per cell from five random fields. Scale bar, 10 μm. B) Cell Counting Kit-8 assay demonstrating viability of OVCAR8 cells after CDDP treatment with indicated peptides. C) SPR sensorgrams showing kinetics of CBP1 binding to SPC25 (left). SPR steady state binding of CBP1 to SPC25 (right). D) Exogenous IP assays for the interactions between SPC25-Flag and RIOK1-HA in HEK293FT cells with escalating doses of CBP1. E) Western blots for β-catenin, RIOK1, and SPC25 expression in OVCAR8 cells treated with indicated peptides. F) Quantified tumor volume from mice subcutaneously inoculated with OVCAR8 cells under indicated treatment after tumor inoculation. *n* = 7 mice per group. G) Kaplan–Meier survival analysis of normal and CBP1-treated nude mice. H) Serum urea, creatinine, alanine aminotransferase, and aspartate aminotransferase levels in normal and CBP1-treated nude mice assessed to evaluate hepatorenal function. I) Relative changes in luminescence signals of intraperitoneal tumors in nude mice receiving indicated treatments in the indicated week (*n* = 7 per group). In (A) and (B), *n* = 3 biological replicates. Error bars represent the means ± SD of independent experiments; ns denotes not significant. In (A) and (H), two-sided Student’s *t* test was used. In (B), one-way repeated-measures analysis of variance was used.

**
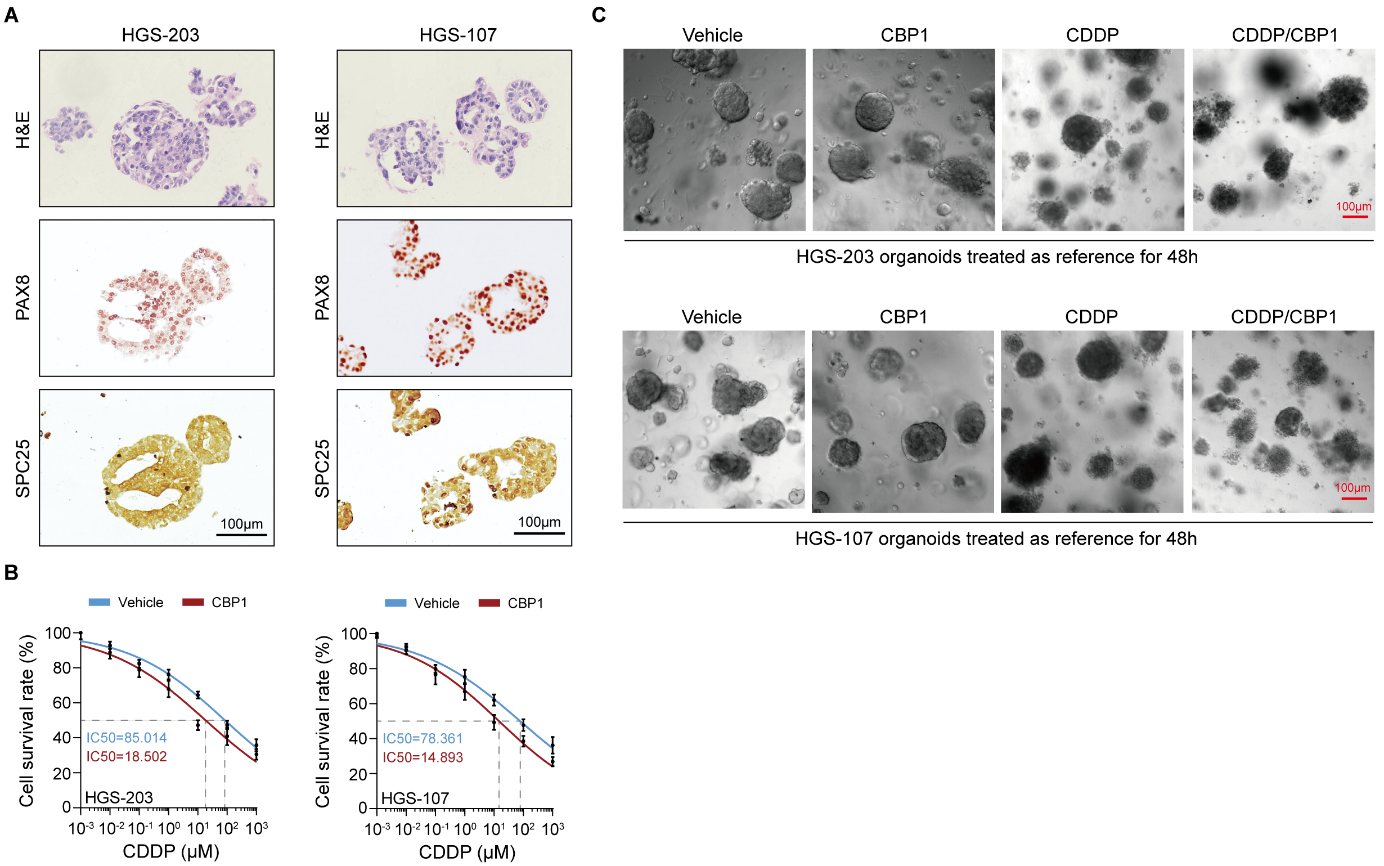
**

**Figure S8.** A) Hematoxylin–eosin (H&E) and IHC staining analysis of PAX8 and SPC25 levels in serial organoids sections. Scale bar, 100 μm. B) Cell Counting Kit-8 assay revealing significantly impaired cell viability in organoids after CDDP treatment in the presence of CBP1. C) Representative images of EOC organoids in the indicated treatment groups. In (B), *n* = 3 biological replicates. In (B), one-way repeated-measures analysis of variance was used.

References

[1]. T. M. Malta, A. Sokolov, A. J. Gentles, T. Burzykowski, L. Poisson, J. N. Weinstein, B. Kaminska, J. Huelsken, L. Omberg, O. Gevaert, A. Colaprico, P. Czerwinska, S. Mazurek, L. Mishra, H. Heyn, A. Krasnitz, A. K. Godwin, A. J. Lazar, N. Cancer Genome Atlas Research, J. M. Stuart, K. A. Hoadley, P. W. Laird, H. Noushmehr, M. Wiznerowicz, Cell 2018, 173 (2), 338.
