# Supplementary material for: Targeting the SPC25/RIOK1/MYH9 Axis to Overcome Tumor Stemness and Platinum Resistance in Epithelial Ovarian Cancer
Source: Adv Sci (Weinh). 2024 Nov 3;11(47):2406688. doi: 10.1002/advs.202406688 (PMC11653702; doi:10.1002/advs.202406688)
Supplement: Supplementary file 2 — Supporting Information [file ADVS-11-2406688-s001.zip › Additional sheet 1 for Table 2.pdf]

Table 2. Provide the complete and correct author list, in order, as it should appear on the article (attach an additional sheet if more space is needed)

Author Name**		Degree/s (e.g. BSc, PhD)	Corr. author (Y/N)	Institutional affiliation	Email address	ORCID iD or Scopus Author ID	Signature*	Date
Given Name†	Family Name							
Lingzhi	Kong	PhD	N	Department of Pathology, S	konglzh@sysucc.or	Not Provided	Kong Lingzhi	01-Oct-20
Yibing	Pan	MA	N	Department of Experimenta	panyb@sysucc.org.	Not Provided	Pan YiBin	01-Oct-20
Pinwei	Deng	MA	N	Department of Experimenta	dengpw@sysucc.or	Not Provided	Deng PingWei	01-Oct-20
Rui	Wang	PhD	N	Department of Experimenta	wangrui@sysucc.or	Not Provided	WangRui	01-Oct-20
Ying	Ouyang	BSc	N	Department of Experimenta	ouyangy1@sysucc.	Not Provided	OY Ying	01-Oct-20
Xiangfu	Chen	BSc	N	Department of Experimenta	chenxf@sysucc.org	Not Provided	Chen X.Fu	01-Oct-20
Jun	Li	PhD	N	Department of Biochemistry	lijun37@mail.sysu.e	Not Provided	Li Jun	01-Oct-20
Zheng	Li	PhD	N	Department of Gynecologic	lizheng@kmmu.edu	Not Provided	Li Zheng	01-Oct-20
Hequn	Zou	PhD	N	School of Medicine, The Ch	zouhequn@cuhk.ec	Not Provided	Zou HeQun	01-Oct-20

*By signing Table 2, individuals and/or representatives of multi-author collaborative or consortia groups confirm that the author list shown here accurately reflect the authorship of the article cited in Table 1
†Include the author's middle initials with the given name, if applicable
**In cases of multi-author collaborative or consortia groups the most appropriate representative or legal guarantor must identify themselves and sign on behalf of the group

No co-author (those remaining, those newly added, those to be removed) or individuals and/or representatives of multi-author collaborative or consortia groups **should sign this form without reading** a) every page of this form in full including the cover page and any attachments, b) the relevant authorship and contributorship policies outlined on the cover page, and c) agreeing with all changes to the author list proposed herein.
