# Supplementary material for: Targeting the SPC25/RIOK1/MYH9 Axis to Overcome Tumor Stemness and Platinum Resistance in Epithelial Ovarian Cancer
Source: Adv Sci (Weinh). 2024 Nov 3;11(47):2406688. doi: 10.1002/advs.202406688 (PMC11653702; doi:10.1002/advs.202406688)
Supplement: Supplementary file 2 — Supporting Information [file ADVS-11-2406688-s001.zip › Additional sheet 2 for Table2.pdf]

Table 2. Provide the complete and correct author list, in order, as it should appear on the article (attach an additional sheet if more space is needed)

Author Name**		Degree/s (e.g. BSc, PhD)	Corr. author (Y/N)	Institutional affiliation	Email address	ORCID iD or Scopus Author ID	Signature*	Date
Given Name†	Family Name							
Yanna	Zhang	PhD	N	Department of Gynecology,	zhangyn@sysucc.o	Not Provided	Zhang yanna	01-Oct-20
Libing	Song	PhD	N	Department of Experimenta	songlb@sysucc.org	0000-0002-53	LibingSong	01-Oct-20

*By signing Table 2, individuals and/or representatives of multi-author collaborative or consortia groups confirm that the author list shown here accurately reflect the authorship of the article cited in Table 1

†Include the author's middle initials with the given name, if applicable

**In cases of multi-author collaborative or consortia groups the most appropriate representative or legal guarantor must identify themselves and sign on behalf of the group

No co-author (those remaining, those newly added, those to be removed) or individuals and/or representatives of multi-author collaborative or consortia groups **should sign this form without reading** a) every page of this form in full including the cover page and any attachments, b) the relevant authorship and contributorship policies outlined on the cover page, and c) agreeing with all changes to the author list proposed herein.
