# Supplementary material for: Targeting the SPC25/RIOK1/MYH9 Axis to Overcome Tumor Stemness and Platinum Resistance in Epithelial Ovarian Cancer
Source: Adv Sci (Weinh). 2024 Nov 3;11(47):2406688. doi: 10.1002/advs.202406688 (PMC11653702; doi:10.1002/advs.202406688)
Supplement: Supplementary file 2 — Supporting Information [file ADVS-11-2406688-s001.zip › Agar Gel.pdf]

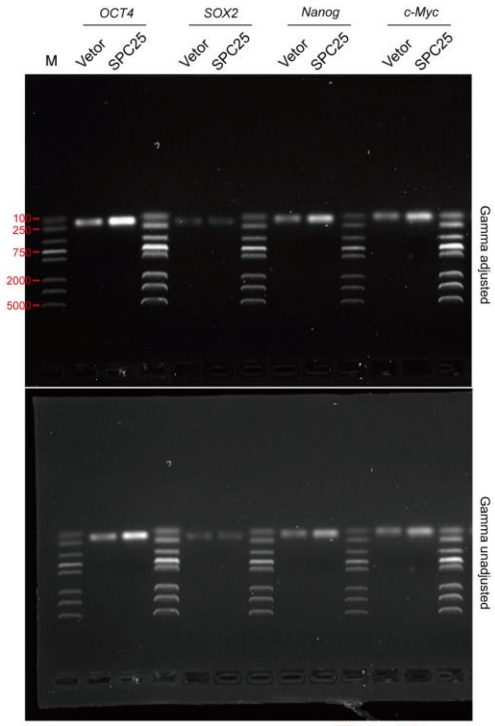

OVCAR3 **(Figure 2K)**

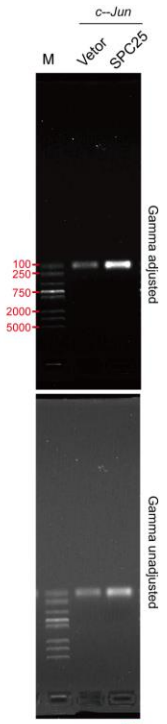

OVCAR3 **(Figure 2K)**

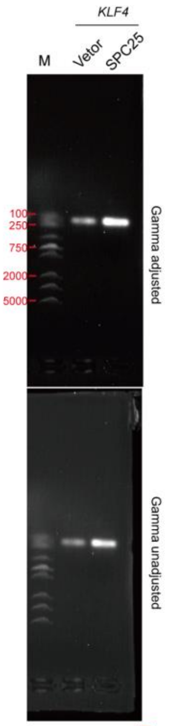

OVCAR3 **(Figure 2K)**

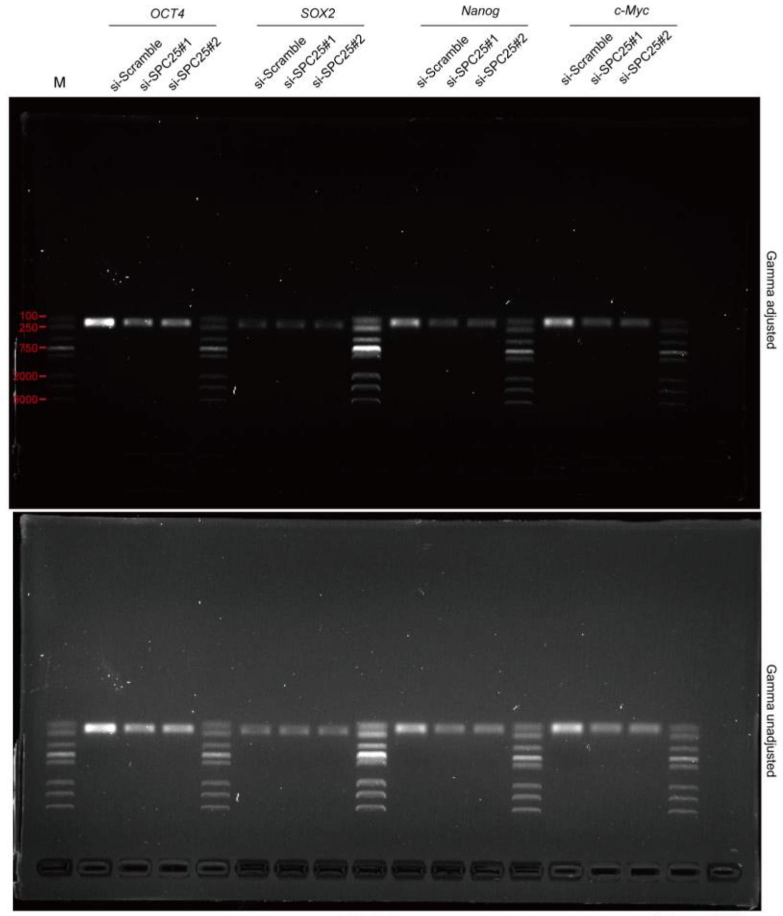

OVCAR8 **(Figure 2K)**

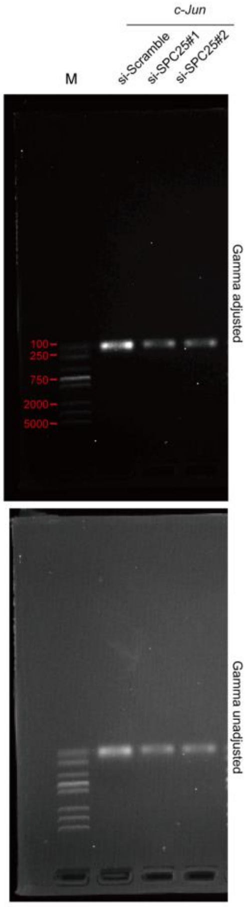

OVCAR8 **(Figure 2K)**

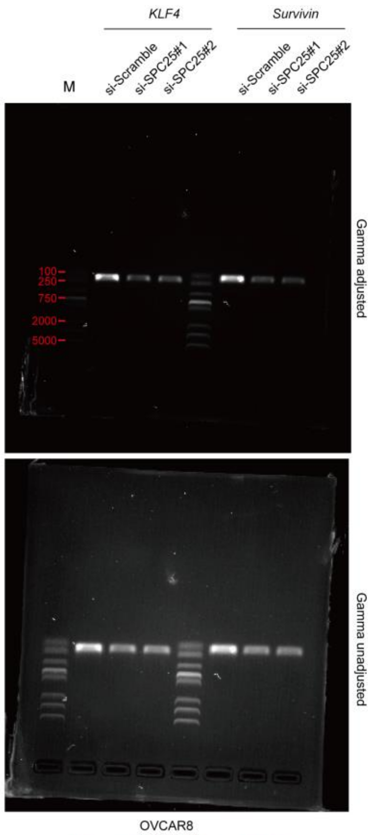

(Figure 2K and Figure S4F)

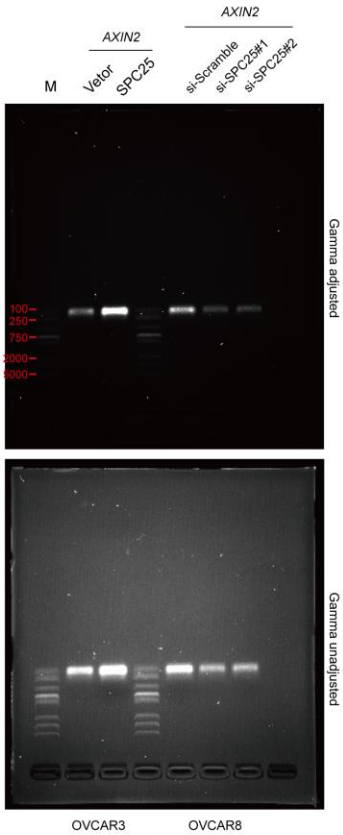

(Figure S4F)

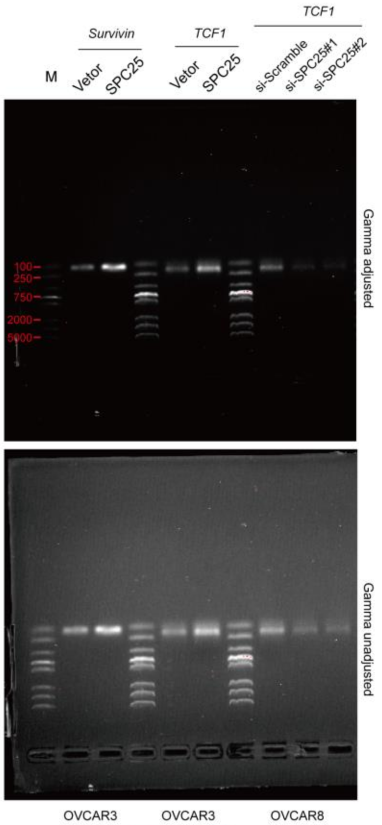

(Figure 2K)

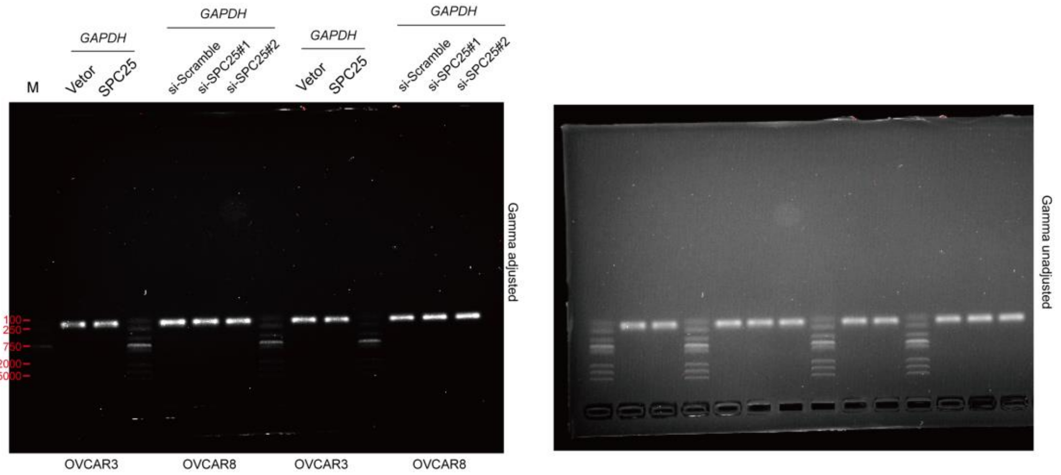

(Figure 2K and Figure S4F)

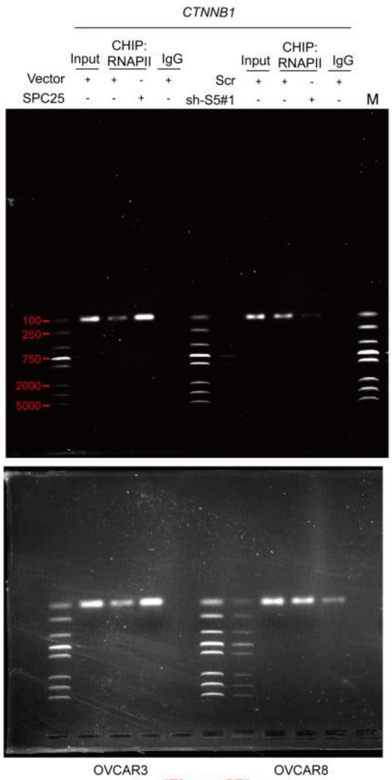

(Figure 2P)

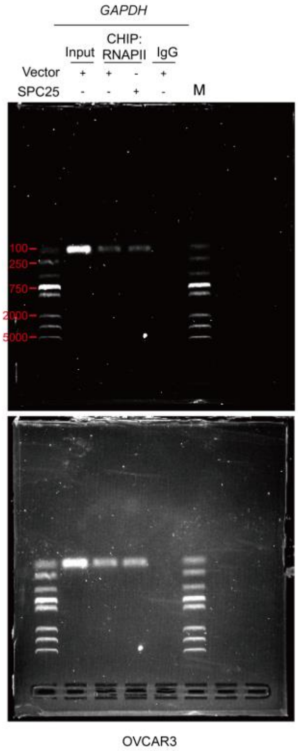

(Figure 2P)

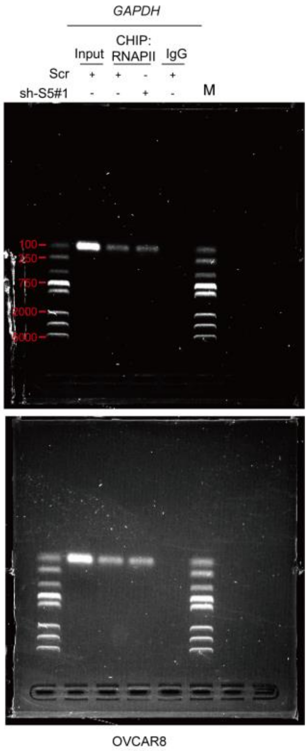

(Figure 2P)

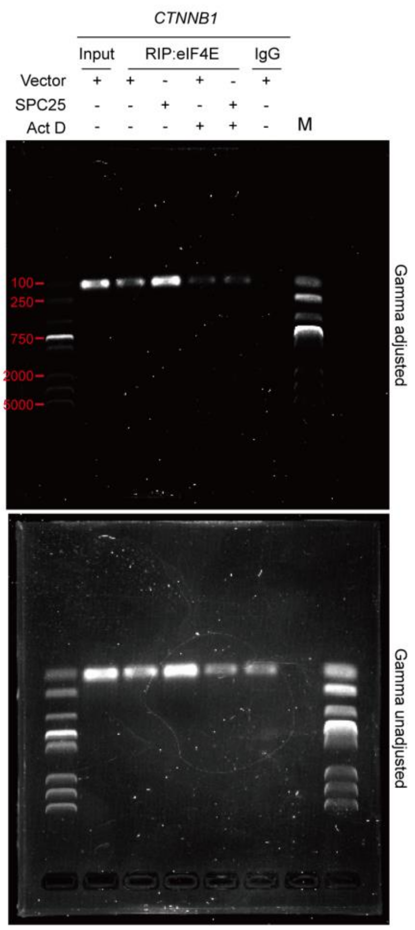

OVCAR3 **(Figure S4L)**

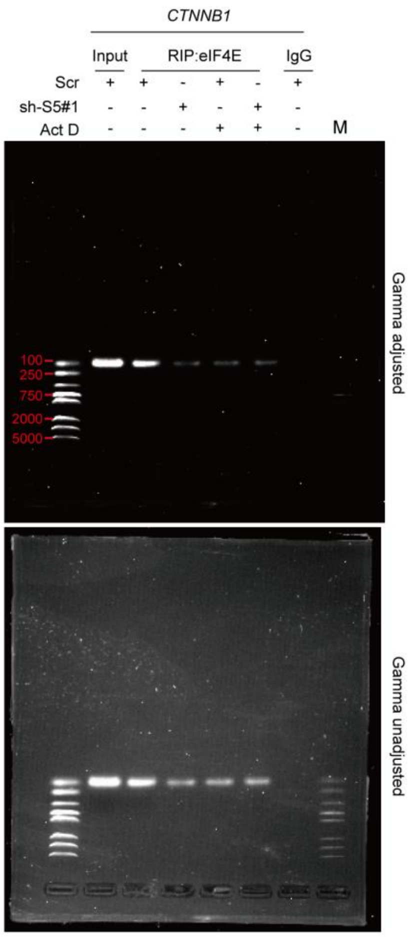

OVCAR8 **(Figure S4M)**

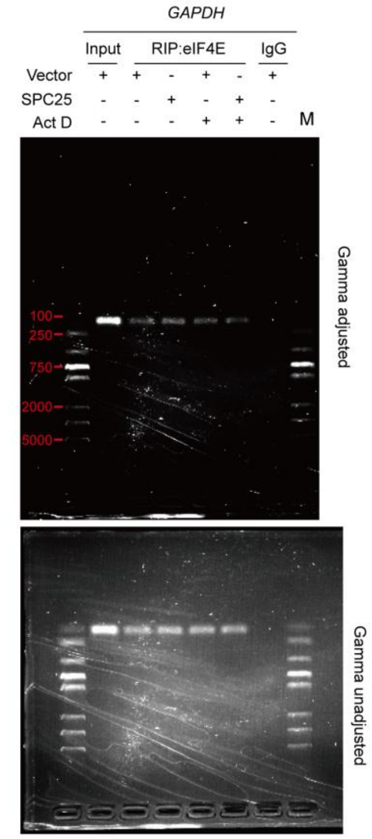

OVCAR3 **(Figure S4L)**

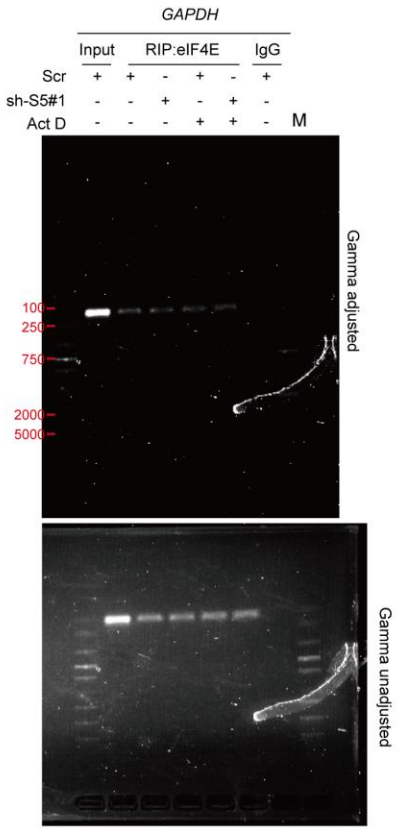

OVCAR8 **(Figure S4M)**
